# Supplementary material for: A machine-learning framework for accelerating spin-lattice relaxation simulations
Source: NPJ Comput Mater. 2025 Mar 6;11(1):62. doi: 10.1038/s41524-025-01547-z (PMC11885155; doi:10.1038/s41524-025-01547-z)
Supplement: Supplementary file 1 — Supplemental material [file 41524_2025_1547_MOESM1_ESM.pdf]

Supplementary Information

Valerio Briganti[1] and Alessandro Lunghi[1,*]

[1] *School of Physics, AMBER and CRANN Institute, Trinity College, Dublin 2, Ireland*
[*] *lunghia@tcd.ie*

1 Machine learning of molecular vibrations - Optimization

1.1 Compound 1

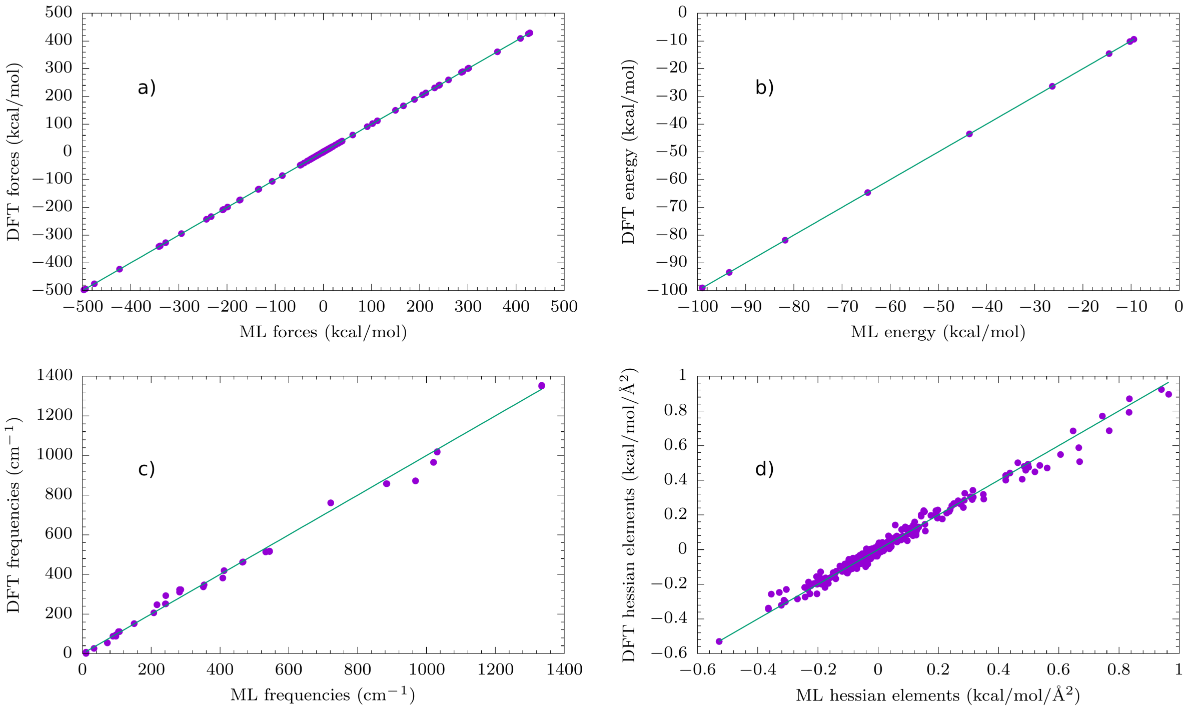

Figure S1: **Parity plots of FF's predictions trained with** $\delta = 5$. a) Forces, b) Energy, c) Vibrational frequency and d) Hessian matrix elements.

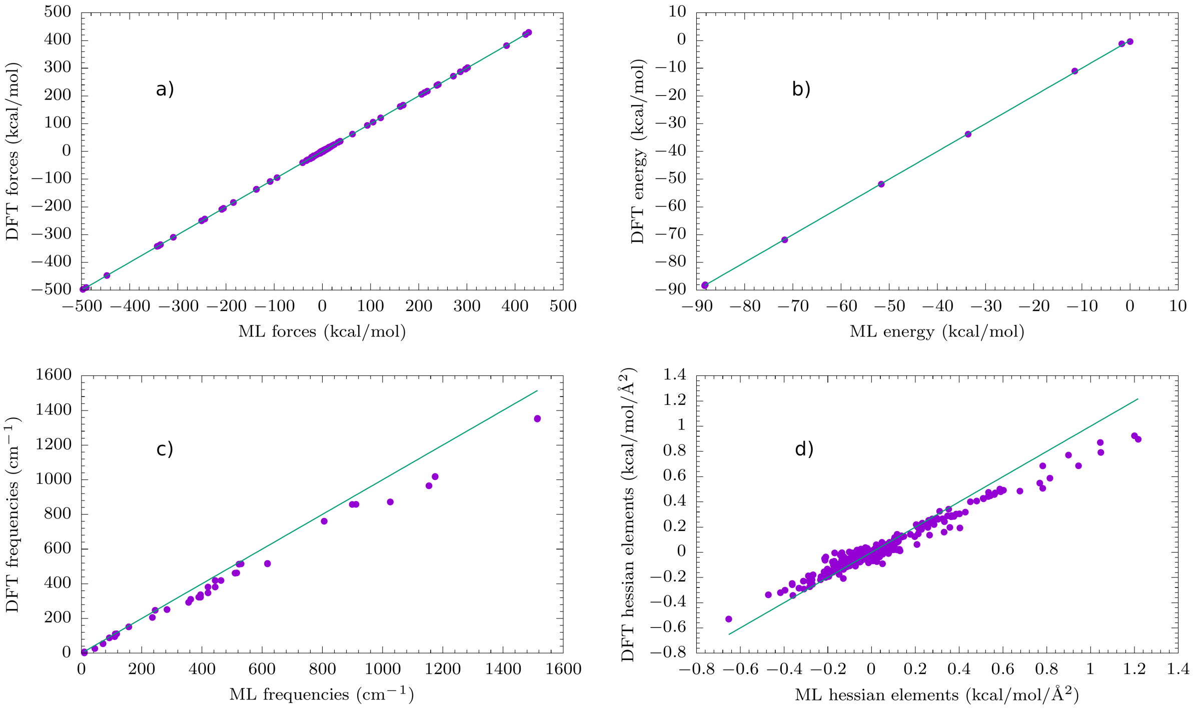

Figure S2: **Parity plots of FF's predictions trained with $\delta = 10$.** a) Forces, b) Energy, c) Vibrational frequency and d) Hessian matrix elements.

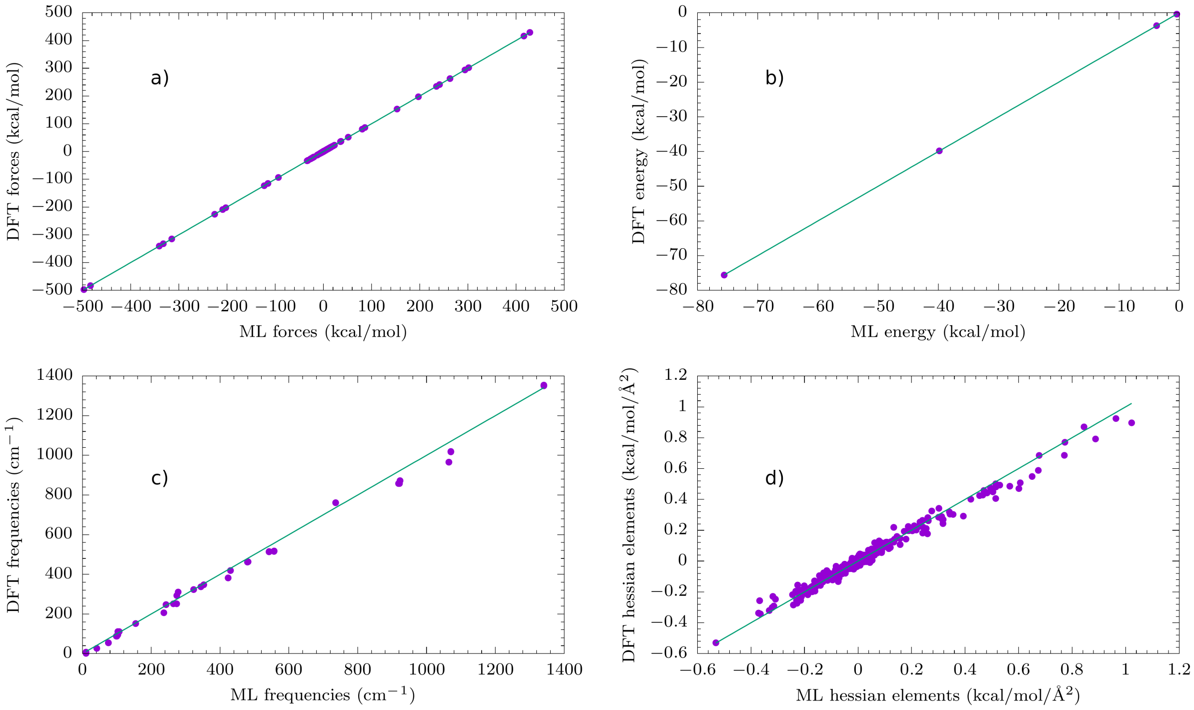

Figure S3: **Parity plots of FF's predictions trained with** $\delta = 20$. a) Forces, b) Energy, c) Vibrational frequency and d) Hessian matrix elements.

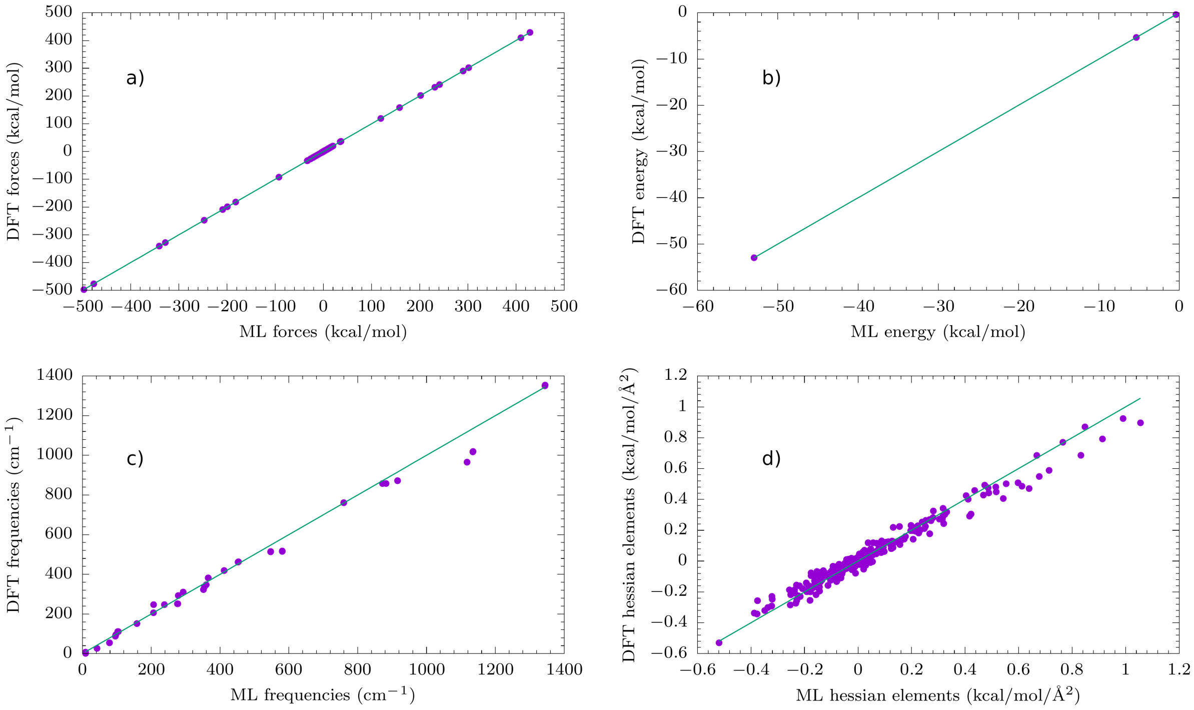

Figure S4: **Parity plots of FF's predictions trained with** $\delta = 30$. a) Forces, b) Energy, c) Vibrational frequency and d) Hessian matrix elements.

1.2 Compound 2

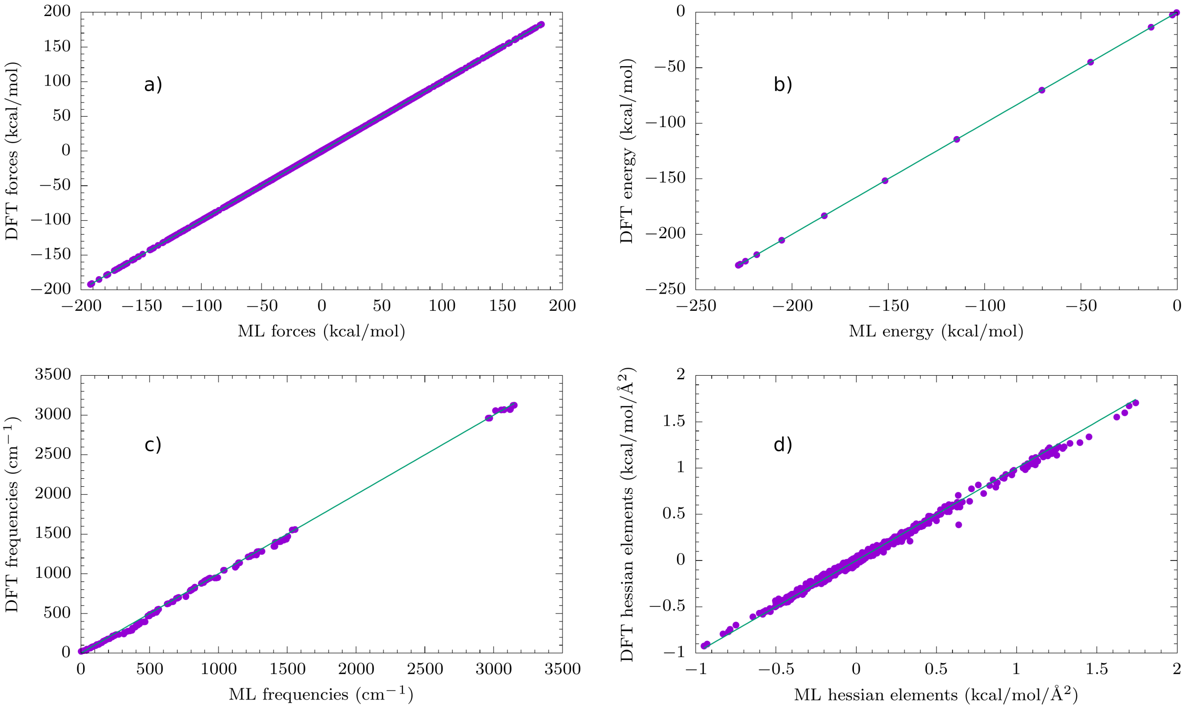

Figure S5: **Parity plots of FF's predictions trained with** $\delta = 5$. a) Forces, b) Energy, c) Vibrational frequency and d) Hessian matrix elements.

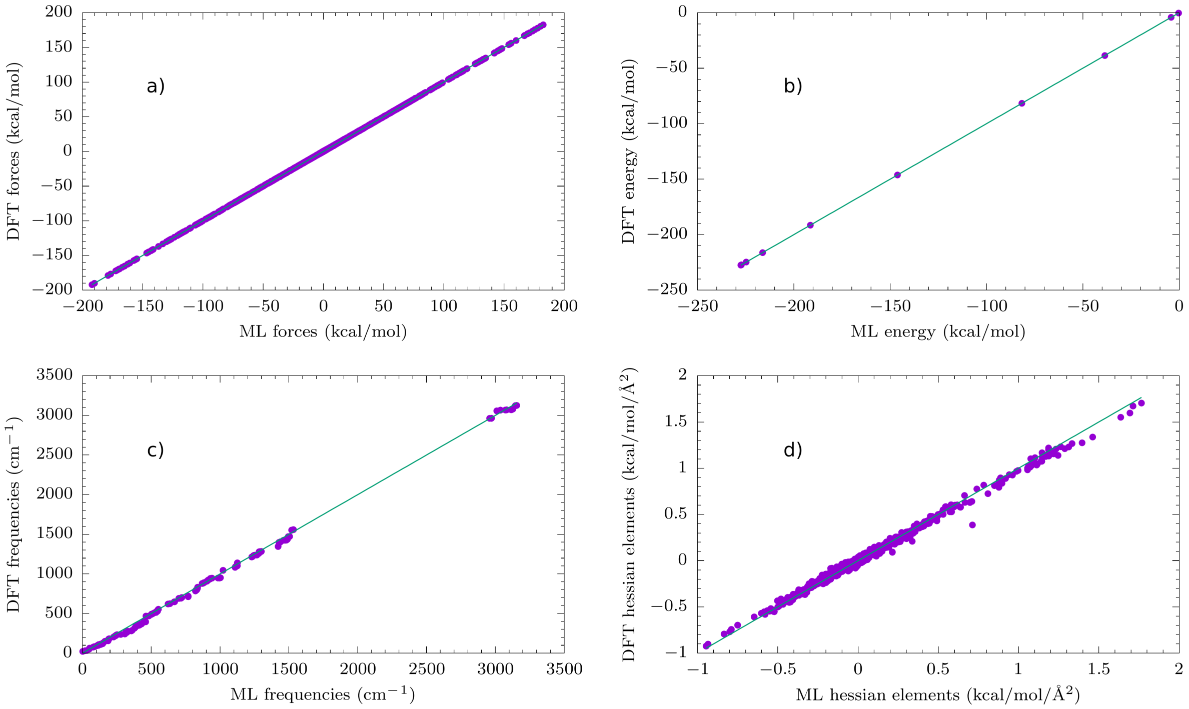

Figure S6: **Parity plots of FF's predictions trained with** $\delta = 10$. a) Forces, b) Energy, c) Vibrational frequency and d) Hessian matrix elements.

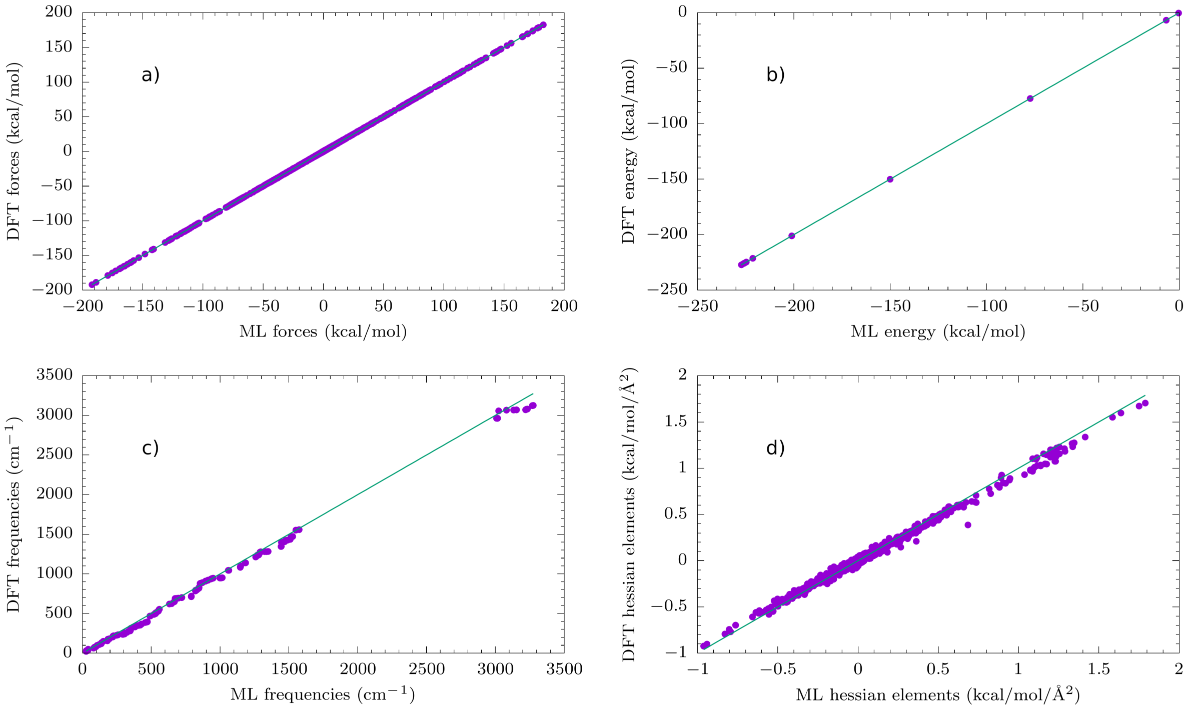

Figure S7: **Parity plots of FF's predictions trained with** $\delta = 20$. a) Forces, b) Energy, c) Vibrational frequency and d) Hessian matrix elements.

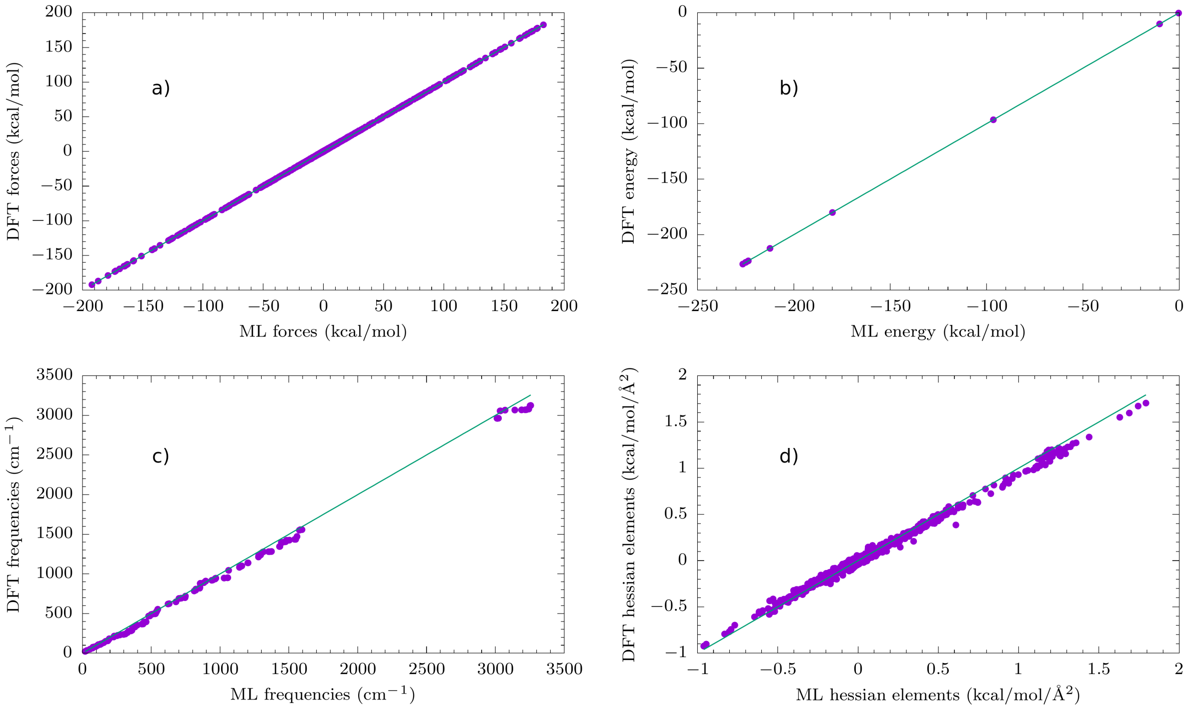

Figure S8: **Parity plots of FF's predictions trained with $\delta = 30$.** a) Forces, b) Energy, c) Vibrational frequency and d) Hessian matrix elements.

1.3 Compound 3

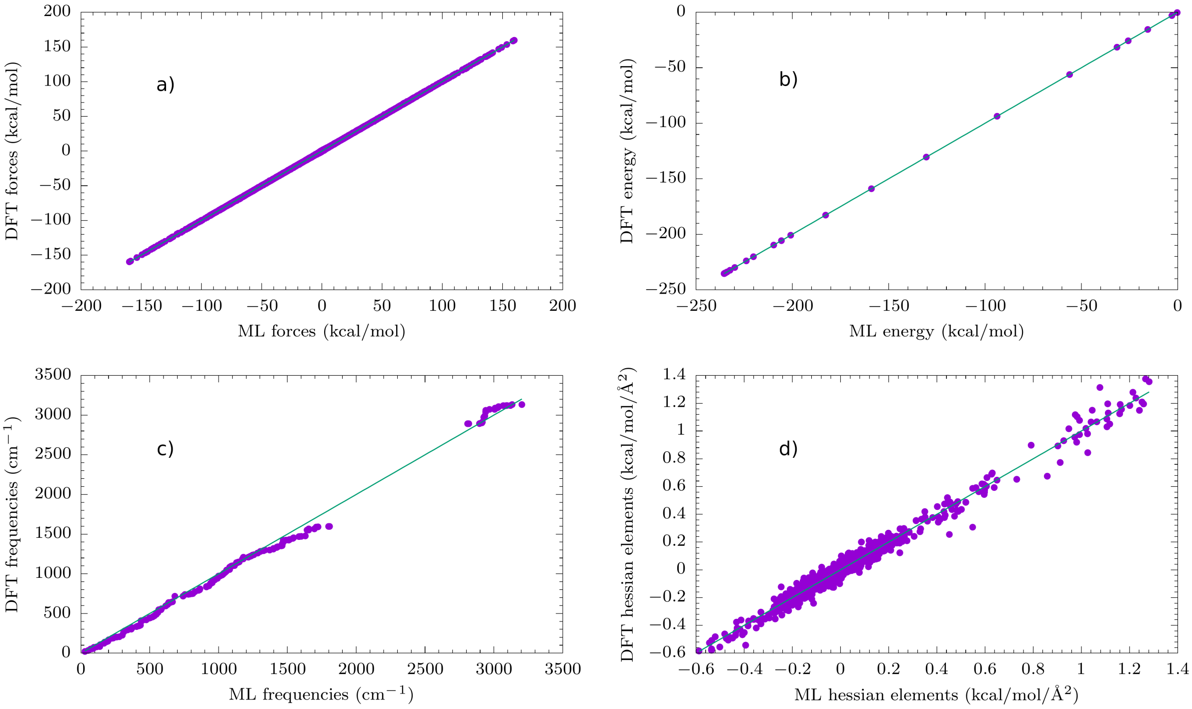

Figure S9: **Parity plots of FF's predictions trained with** $\delta = 5$. a) Forces, b) Energy, c) Vibrational frequency and d) Hessian matrix elements.

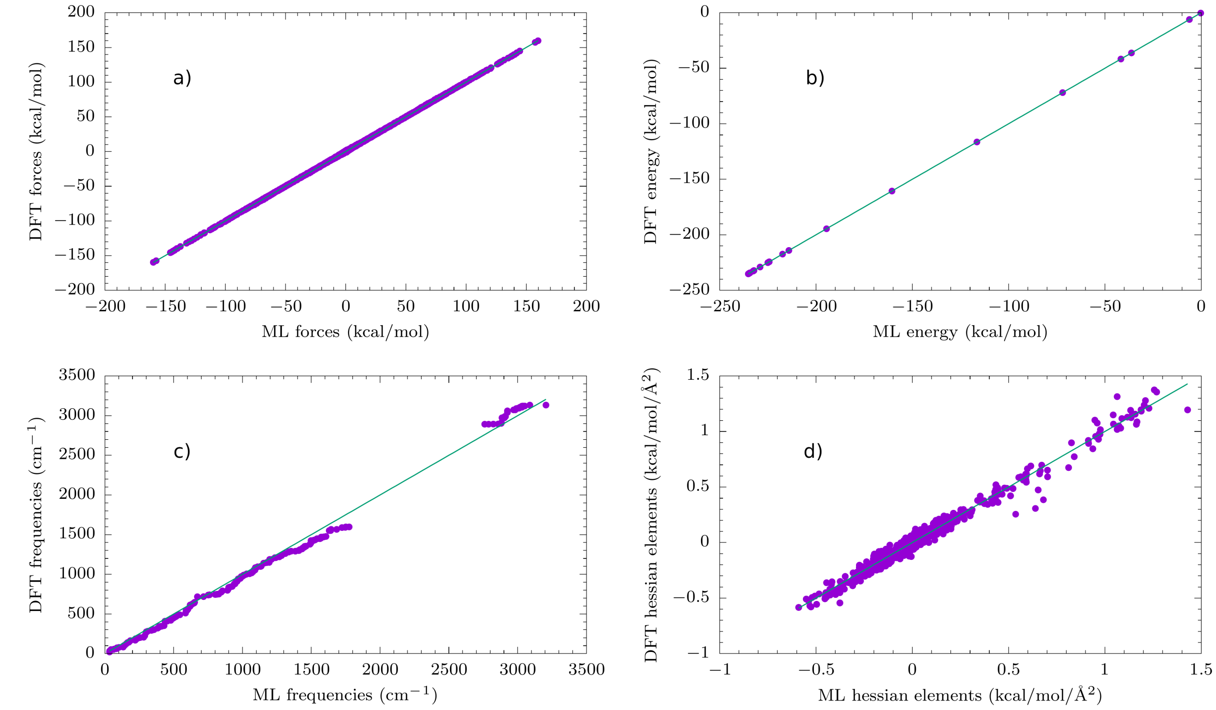

Figure S10: **Parity plots of FF's predictions trained with** $\delta = 10$. a) Forces, b) Energy, c) Vibrational frequency and d) Hessian matrix elements.

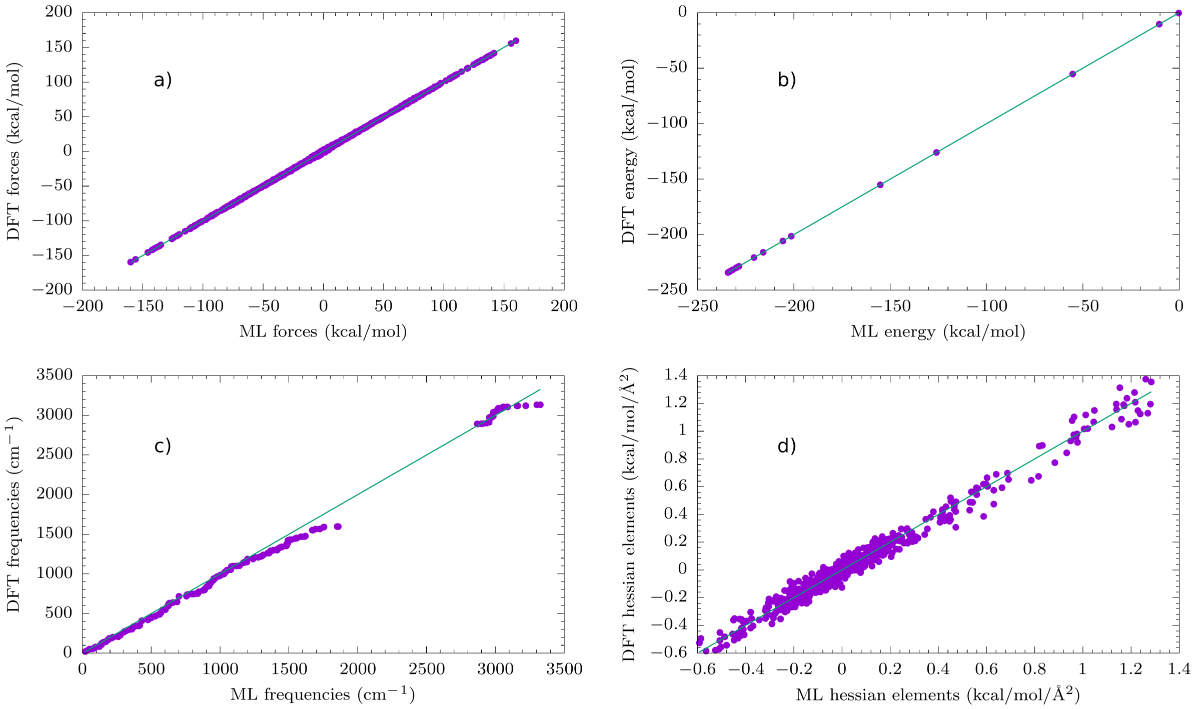

Figure S11: **Parity plots of FF's predictions trained with** $\delta = 20$. a) Forces, b) Energy, c) Vibrational frequency and d) Hessian matrix elements.

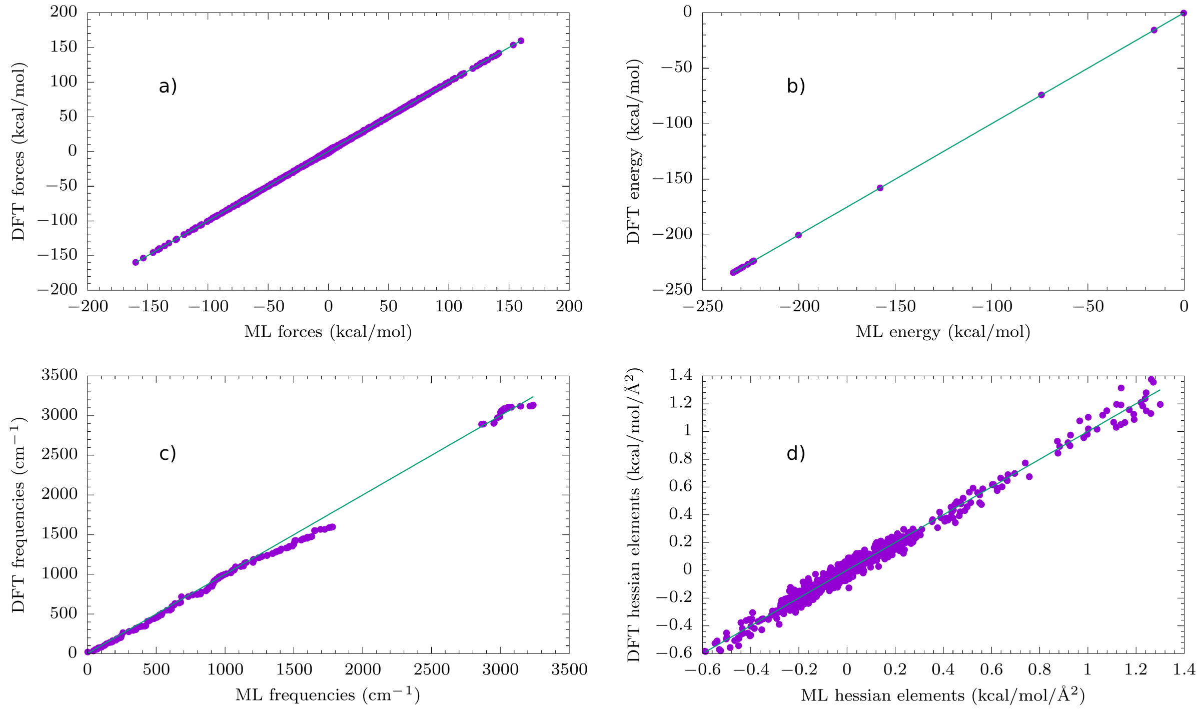

Figure S12: **Parity plots of FF's predictions trained with** $\delta = 30$. a) Forces, b) Energy, c) Vibrational frequency and d) Hessian matrix elements.

2 Machine learning of molecular vibrations - MD @50 K

2.1 Compound 1

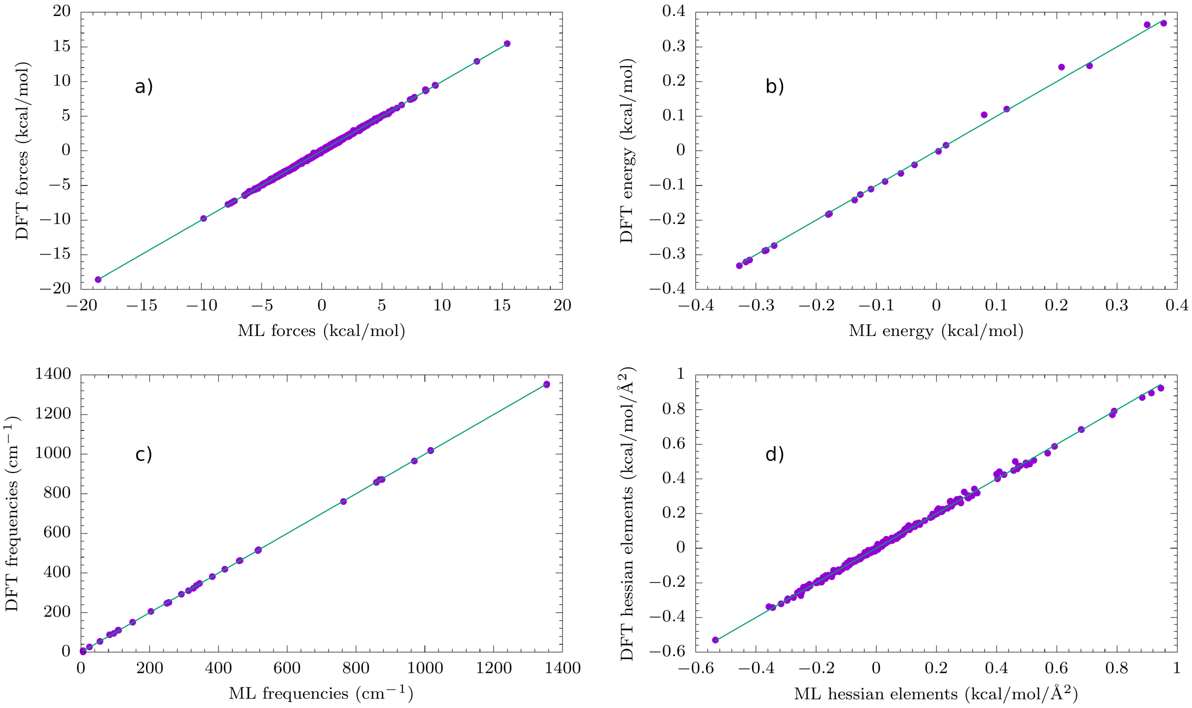

Figure S13: **Parity plots of FF's predictions trained with** $\delta = 2.5$. a) Forces, b) Energy, c) Vibrational frequency and d) Hessian matrix elements.

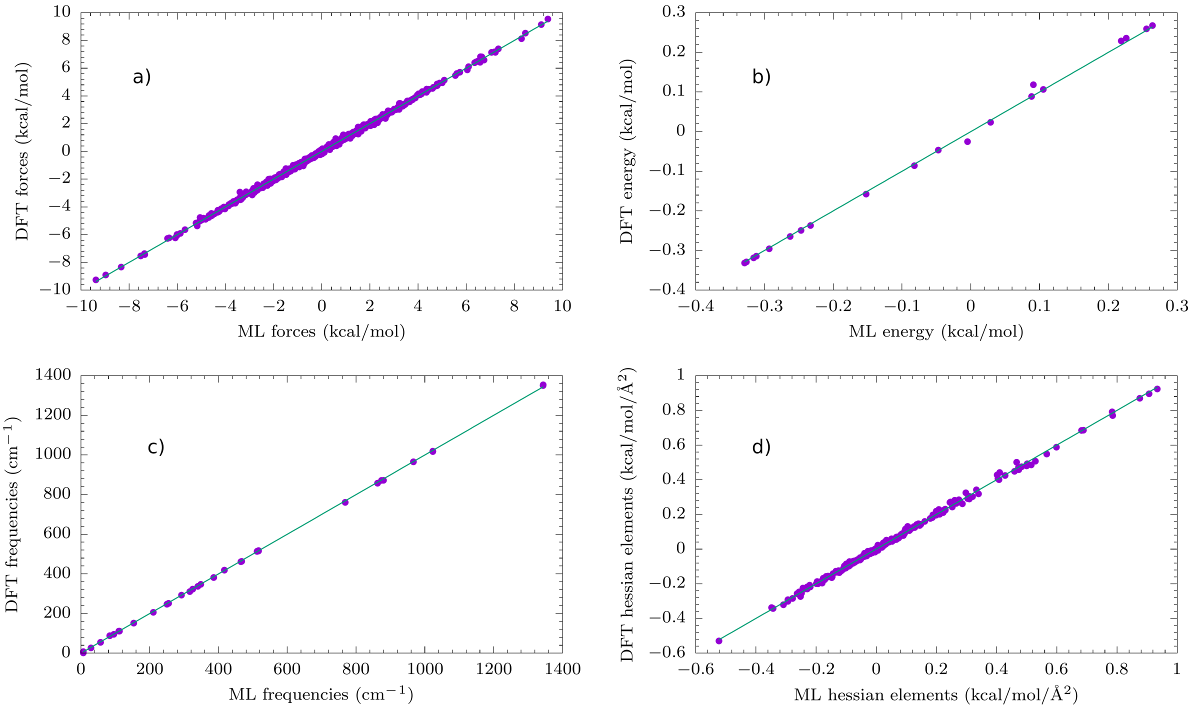

Figure S14: **Parity plots of FF's predictions trained with** $\delta = 2.75$. a) Forces, b) Energy, c) Vibrational frequency and d) Hessian matrix elements.

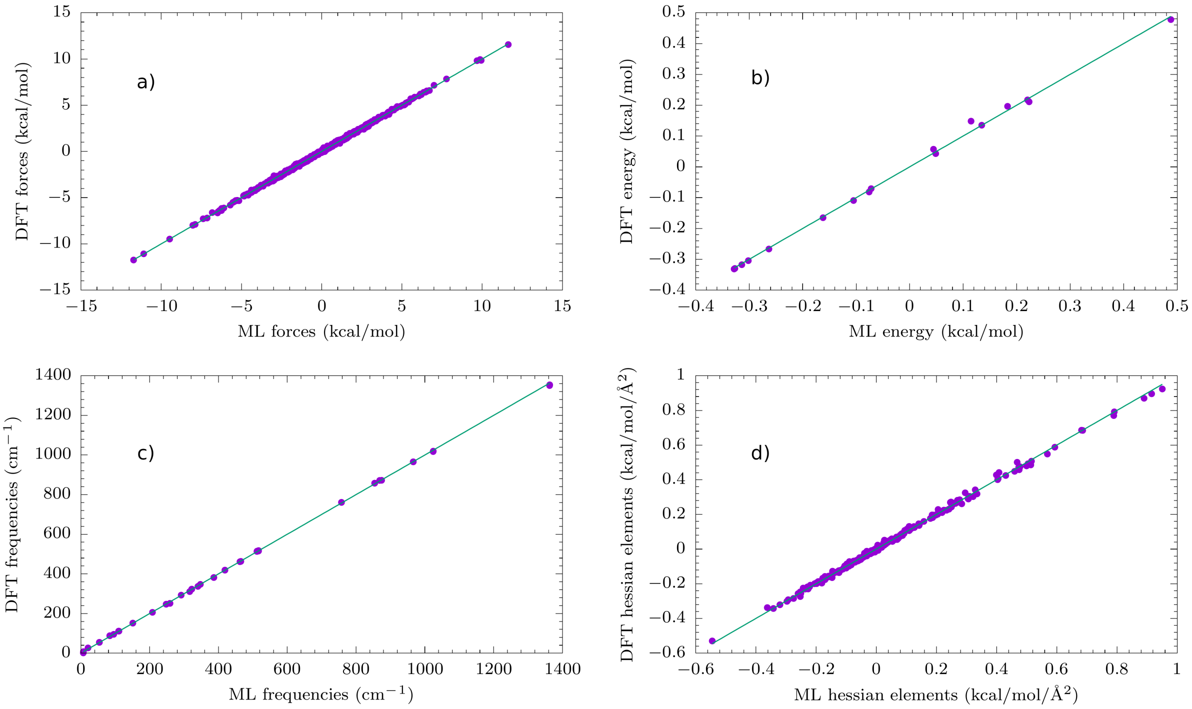

Figure S15: **Parity plots of FF's predictions trained with** $\delta = 3$. a) Forces, b) Energy, c) Vibrational frequency and d) Hessian matrix elements.

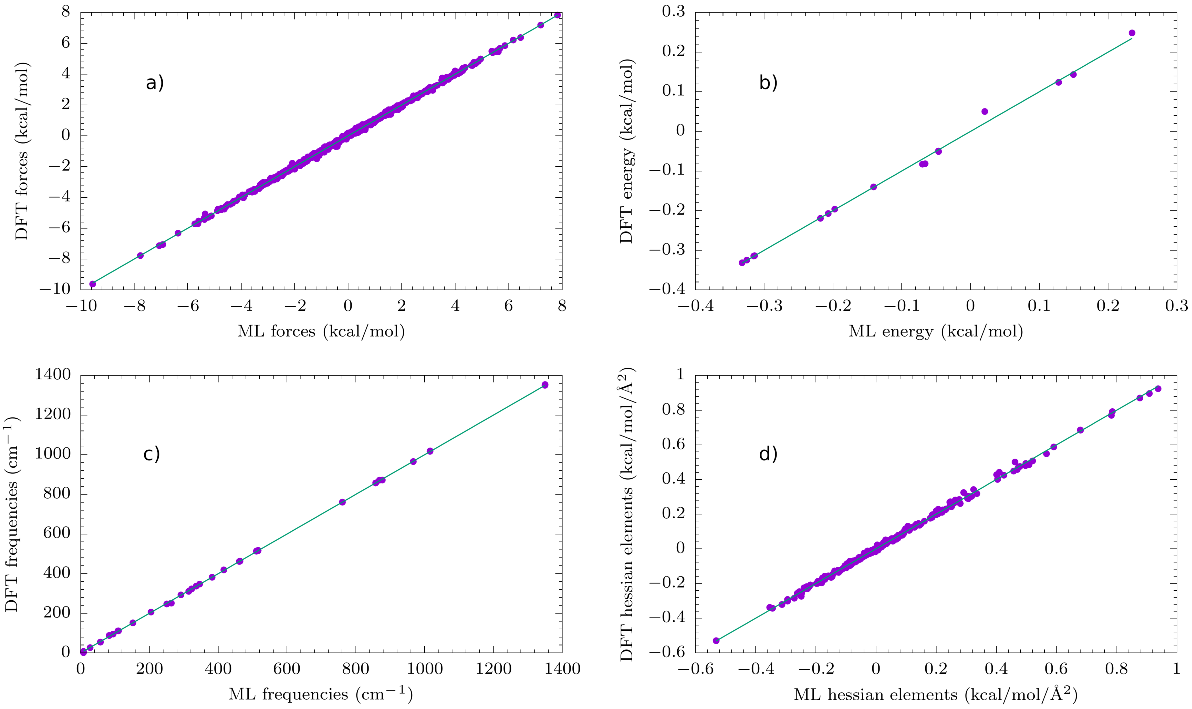

Figure S16: **Parity plots of FF's predictions trained with** $\delta = 3.25$. a) Forces, b) Energy, c) Vibrational frequency and d) Hessian matrix elements.

2.2 Compound 2

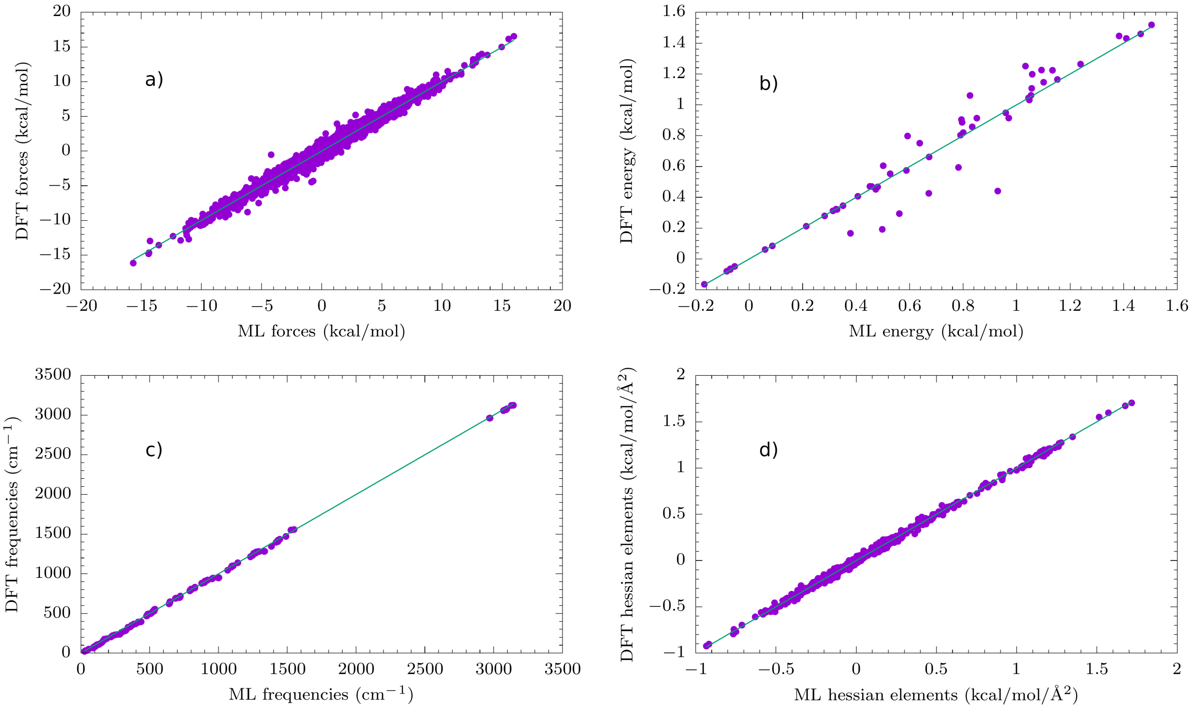

Figure S17: **Parity plots of FF's predictions trained with** $\delta = 2.5$. a) Forces, b) Energy, c) Vibrational frequency and d) Hessian matrix elements.

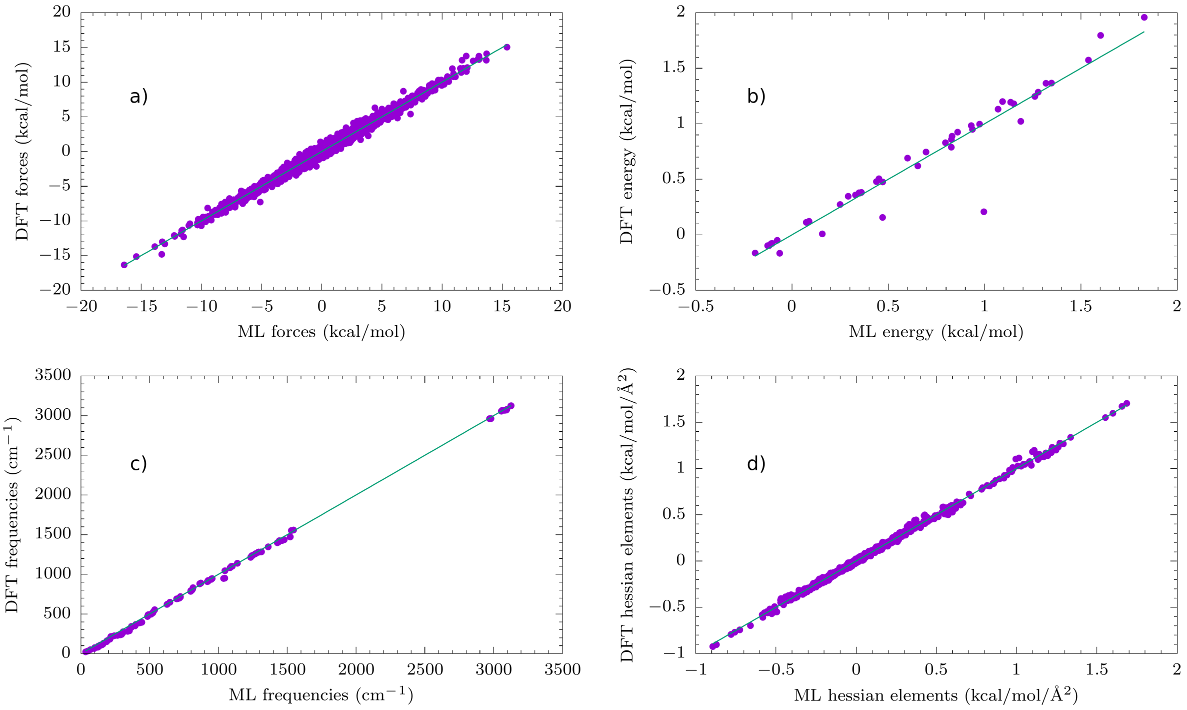

Figure S18: **Parity plots of FF's predictions trained with** $\delta = 2.75$. a) Forces, b) Energy, c) Vibrational frequency and d) Hessian matrix elements.

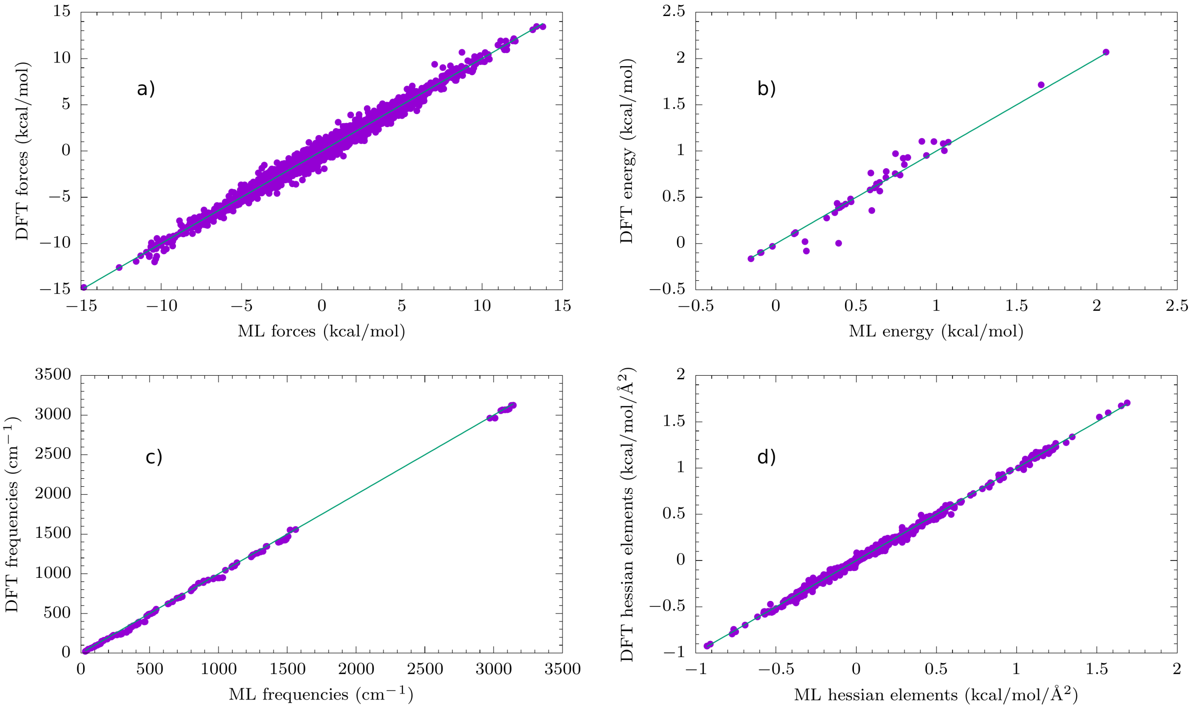

Figure S19: **Parity plots of FF's predictions trained with $\delta = 3$.** a) Forces, b) Energy, c) Vibrational frequency and d) Hessian matrix elements.

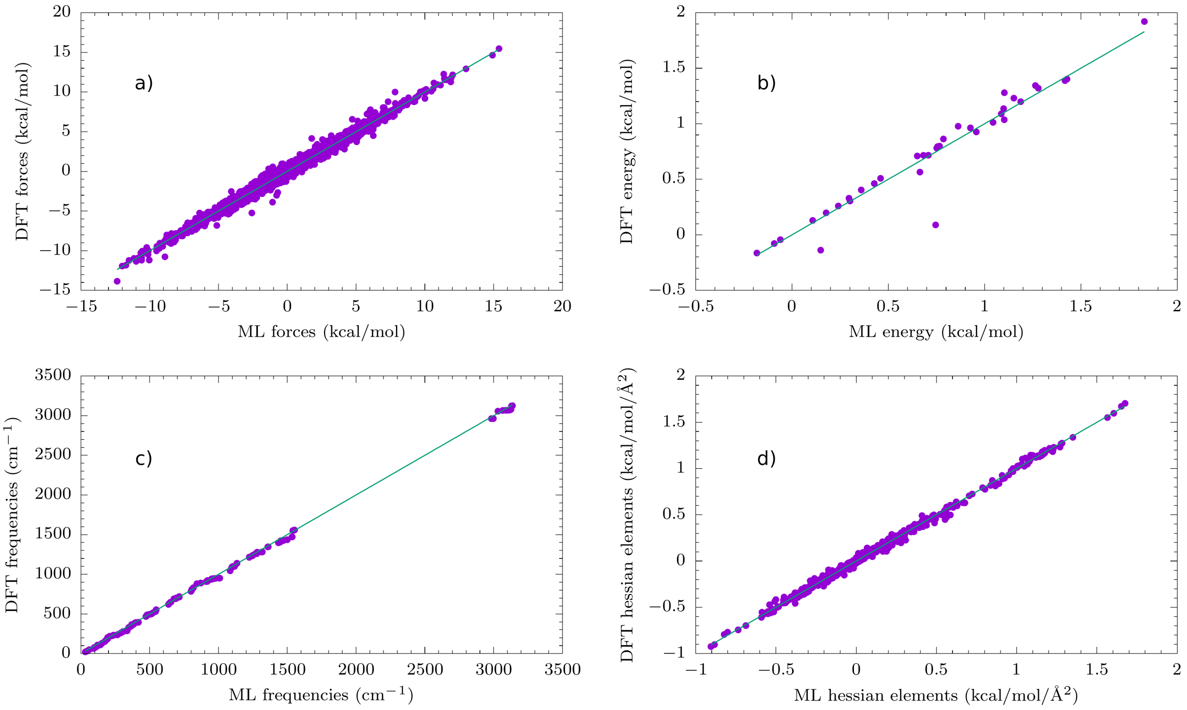

Figure S20: **Parity plots of FF's predictions trained with $\delta = 3.25$.** a) Forces, b) Energy, c) Vibrational frequency and d) Hessian matrix elements.

2.3 Compound 3

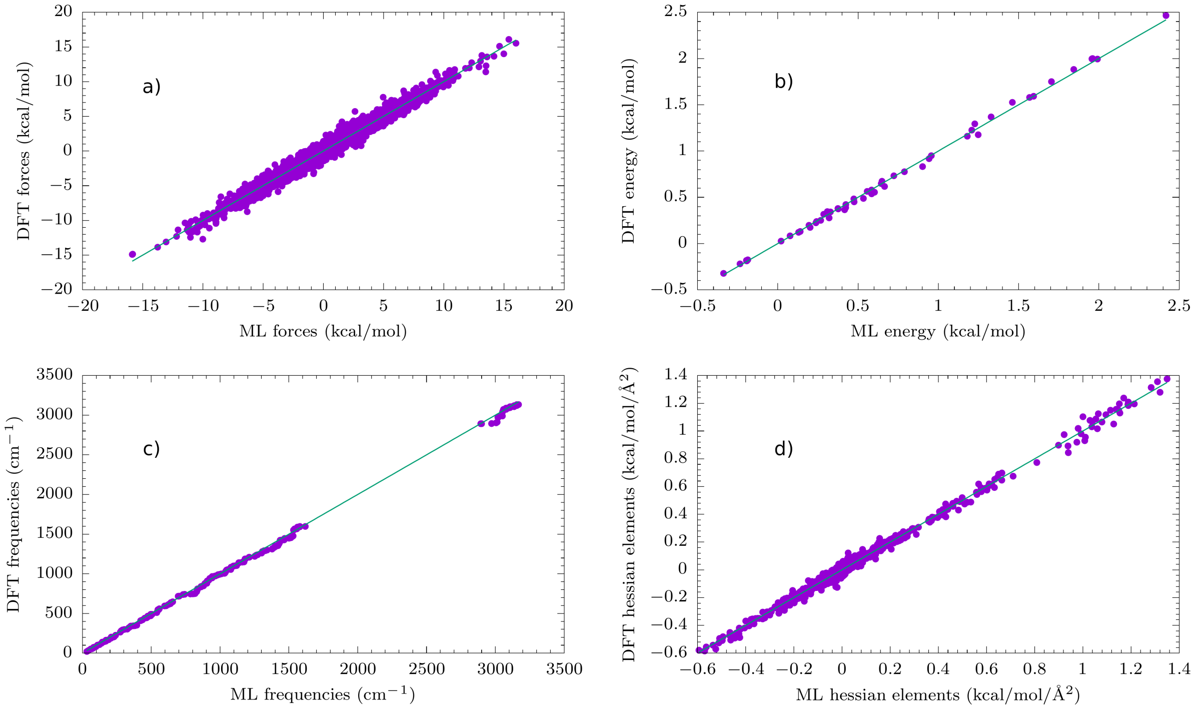

Figure S21: **Parity plots of FF's predictions trained with** $\delta = 2.5$. a) Forces, b) Energy, c) Vibrational frequency and d) Hessian matrix elements.

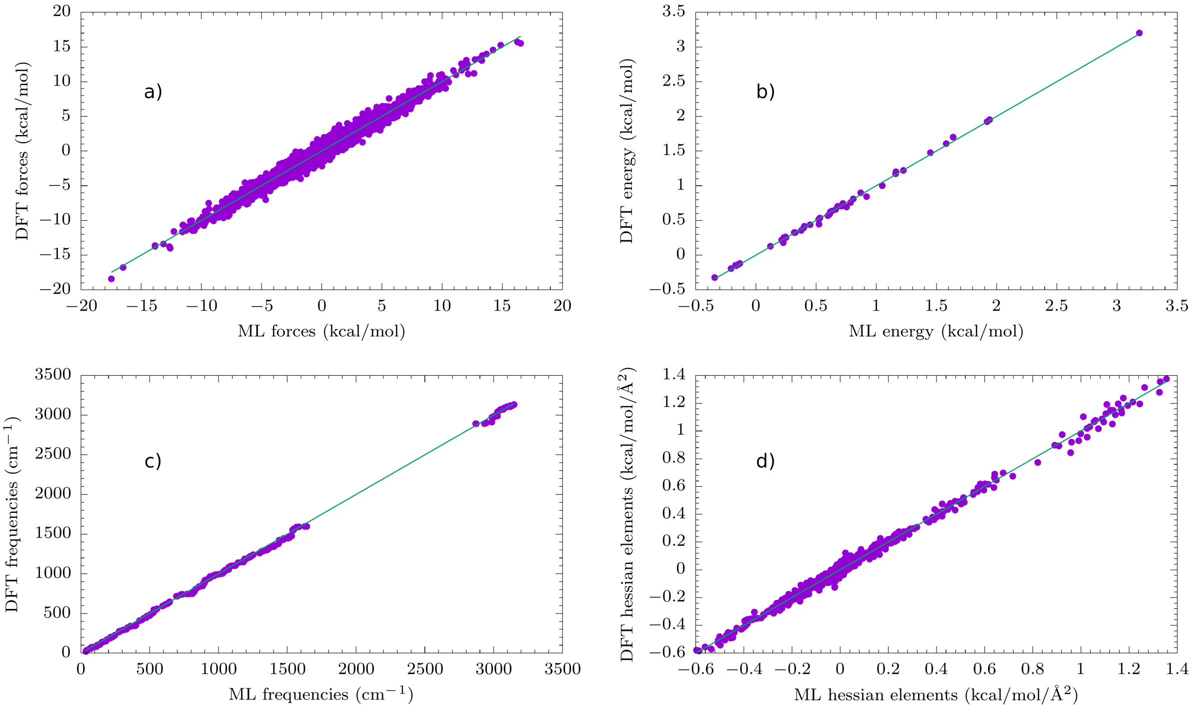

Figure S22: **Parity plots of FF's predictions trained with** $\delta = 2.75$. a) Forces, b) Energy, c) Vibrational frequency and d) Hessian matrix elements.

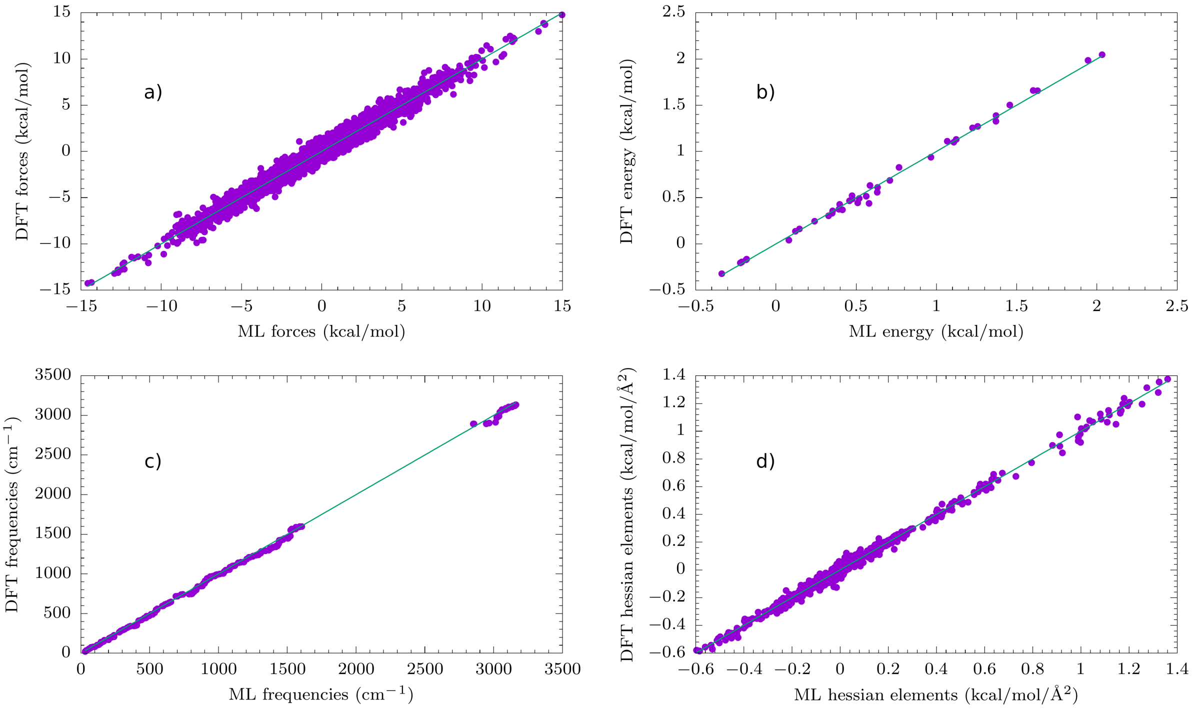

Figure S23: **Parity plots of FF's predictions trained with** $\delta = 3$. a) Forces, b) Energy, c) Vibrational frequency and d) Hessian matrix elements.

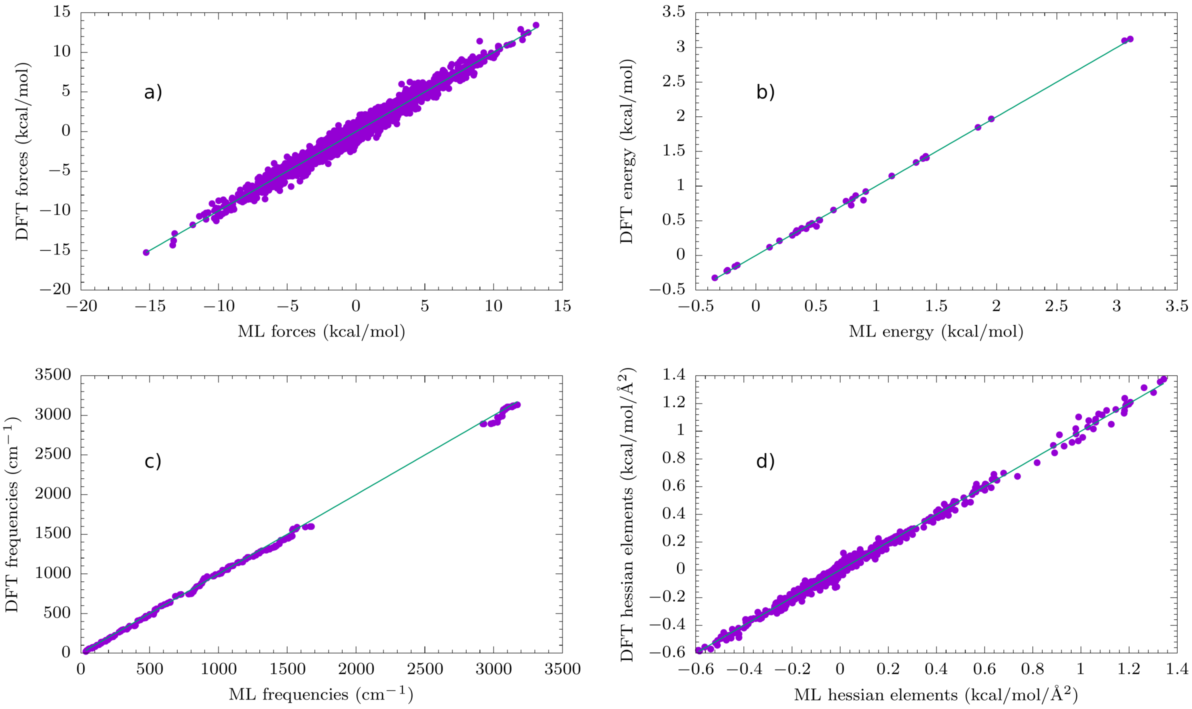

Figure S24: **Parity plots of FF's predictions trained with** $\delta = 3.25$. a) Forces, b) Energy, c) Vibrational frequency and d) Hessian matrix elements.

Table S1: **Optimization results for the selected compounds** The RMSE for phonons and hessians are reported in cm^{-1} and kcal/mol/Å^2. The training set size (TSS) selected by the active learning algorithm is also reported for different values of the threshold parameter δ. The efficiency factor (E.F) is reported as a percentage.

**Compound**	δ	**E.F.**	**TSS**	**RMSE Phonons**	**RMSE Hessian**
**1**	5	93%	9	30.8	0.013
	10	94%	8	57.07	0.032
	20	97%	4	29.3	0.014
	30	98%	3	42.17	0.019
**2**	5	96%	14	23.54	0.007
	10	97%	10	25.61	0.008
	20	97%	10	38	0.009
	30	97%	9	38.3	0.009
**3**	5	93%	26	52.12	0.011
	10	95%	21	59.6	0.011
	20	95%	19	52.6	0.012
	30	96%	15	51.2	0.011

Table S2: **Phonon results after molecular dynamics at 50 K** The RMSE for phonons and hessians are reported in cm^{-1} and kcal/mol/Å^2. The efficiency factor (E.F) is reported as a percentage. The training set size (TSS) selected by the active learning algorithm is also reported for different values of the threshold parameter δ.

**Compound**	δ	**E.F.**	**TSS**	**RMSE Phonons**	**RMSE Hessian**
**1**	2.5	80%	22	2.47	0.004
	2.75	82%	20	3.63	0.004
	3	84%	17	3.89	0.004
	3.25	85%	16	3.55	0.004
**2**	2.5	81%	54	14.8	0.005
	2.75	84%	44	21.8	0.005
	3	85%	43	21.15	0.005
	3.25	87%	36	19.5	0.005
**3**	2.5	83%	53	23.29	0.006
	2.75	85%	44	21.9	0.006
	3	85%	41	24.1	0.007
	3.25	87%	38	28.5	0.007

3 Machine learning of spin-phonon coupling

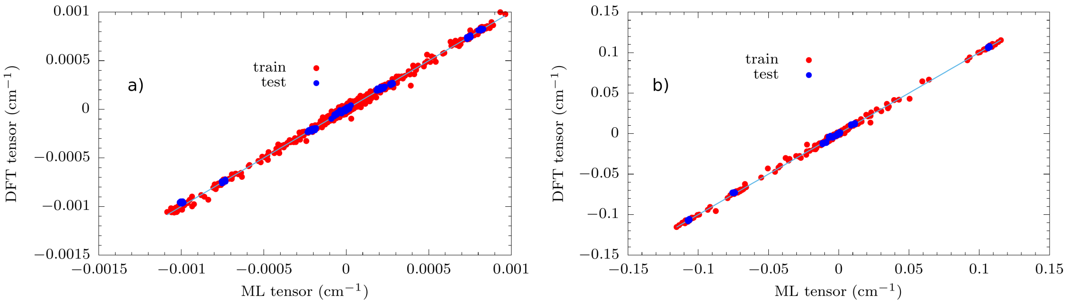

Figure S25: **Fit tensors**. Training and test error for the fit of the spherical tensor components in which the tesseral functions are decomposed. a) $l = 4$, b) $l = 6$

Table S3: **RMSE on spin Hamiltonian tensor fit** The unit of measure is cm^{-1}. The results for **3** are reported for the different order of the tensor $l = 2, 4, 6$, in order.

Comp	RMSE Train	RMSE Test
**1**	0.36	0.19
**2**	0.18	0.10
**3**	$3.5 \cdot 10^{-2}$<br>$1.2 \cdot 10^{-3}$<br>$2.0 \cdot 10^{-5}$	$2.0 \cdot 10^{-2}$<br>$9.5 \cdot 10^{-4}$<br>$1.0 \cdot 10^{-5}$

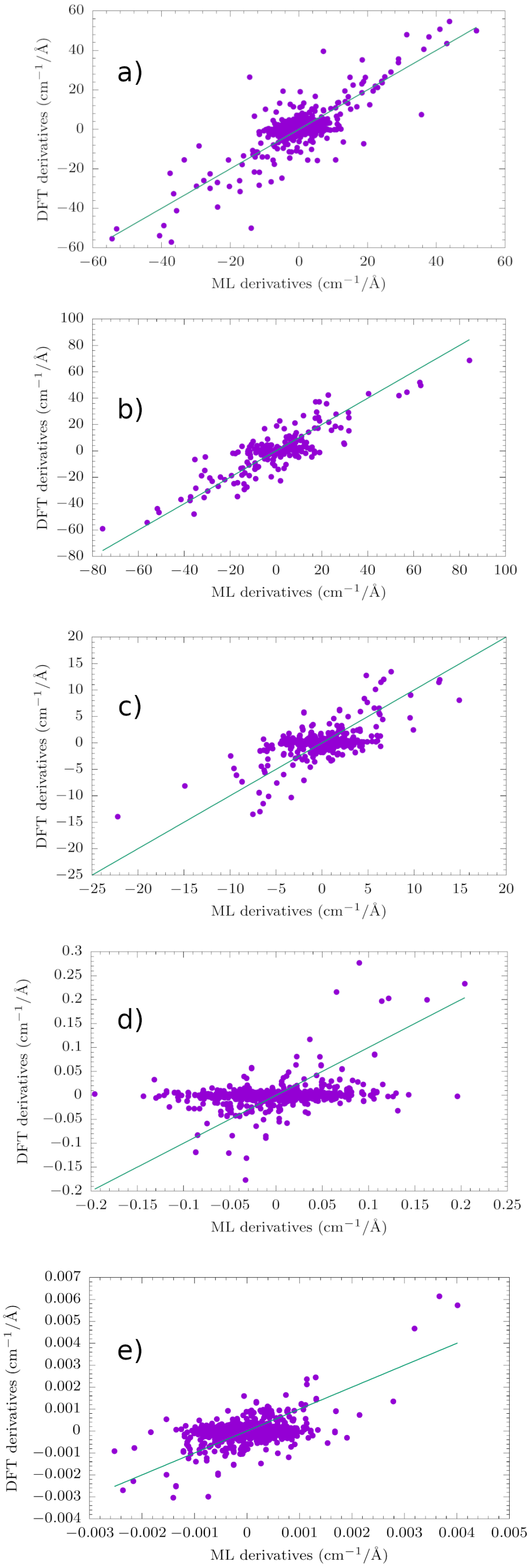

Figure S26: **Derivatives of the spin Hamiltonian $\partial \hat{H}_0/\partial X_i$ with ML tensorial predictions** a) Compound **1**, b) Compound **2**, c) Compound **3** ($l = 2$), d) Compound **3** ($l = 4$), e) Compound **3** ($l = 6$)

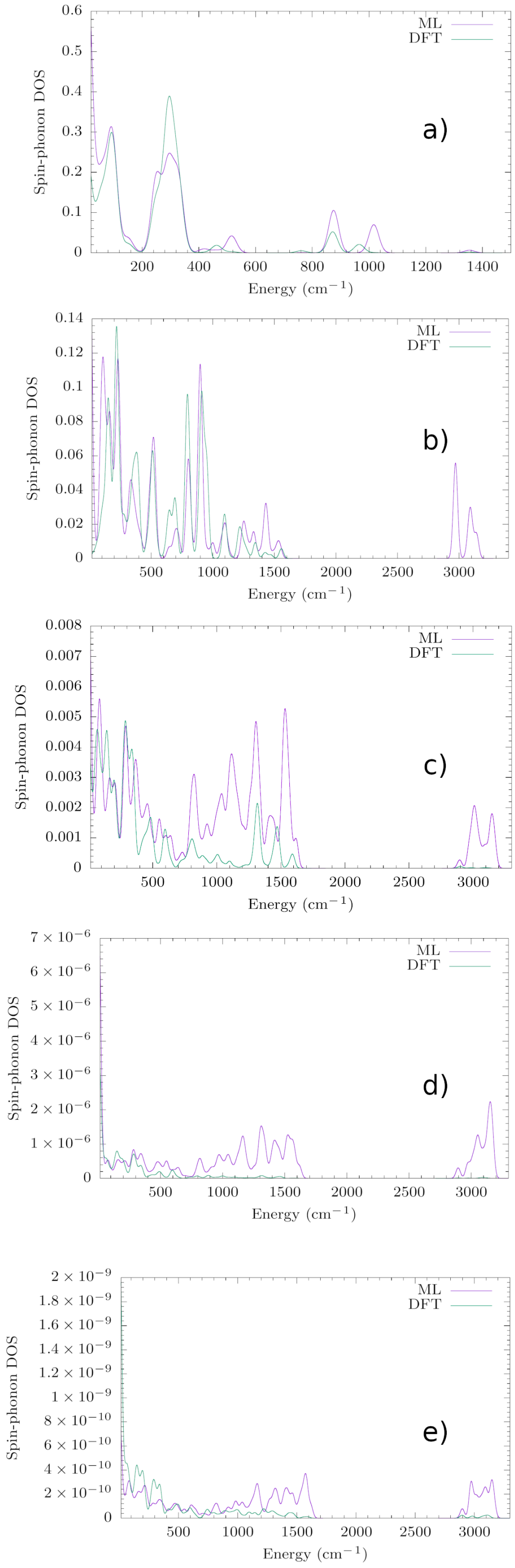

Figure S27: **Spin-phonon densities.** Comparison between the spin-phonon densities calculated with ML predicted Spin Hamiltonian tensors and phonons and with a full quantum mechanical approach. a) Compound **1**, b) Compound **2**, c) Compound **3** ($l = 2$), d) Compound **3** ($l = 4$), e) Compound **3** ($l = 6$) .

4 Machine learning the full spin-potential energy surface

4.1 Compound 1

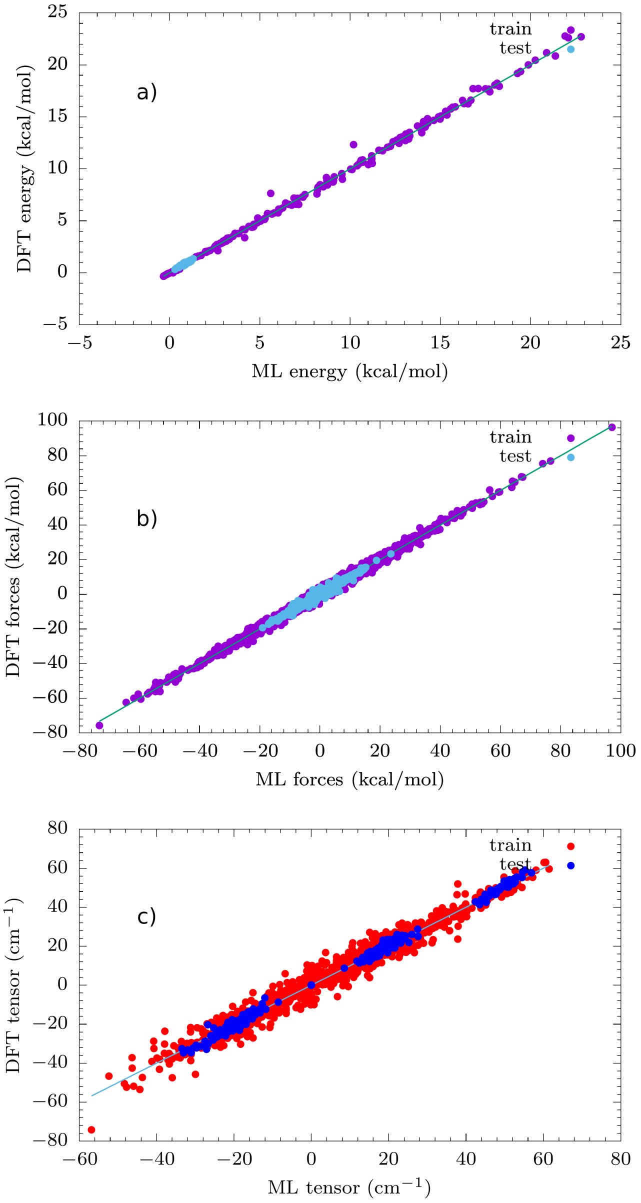

Figure S28: **Parity plots for predictions at T=25 K.** a) Energy, b) Forces, c) Tensorial components

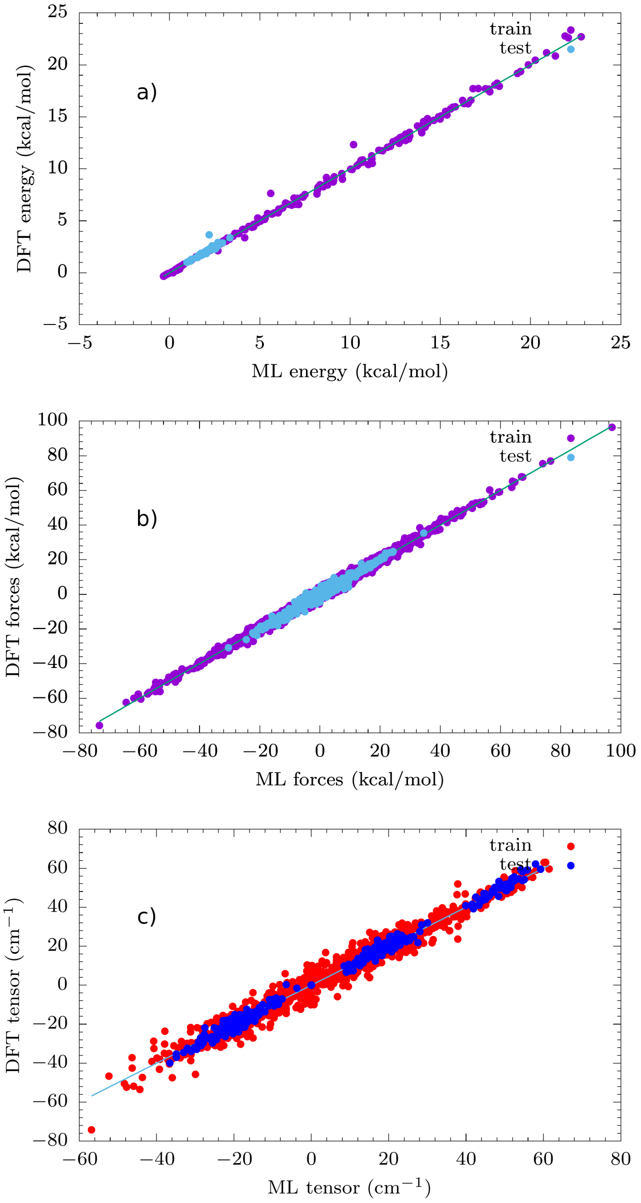

Figure S29: **Parity plots for predictions at T=50 K.** a) Energy, b) Forces, c) Tensorial components

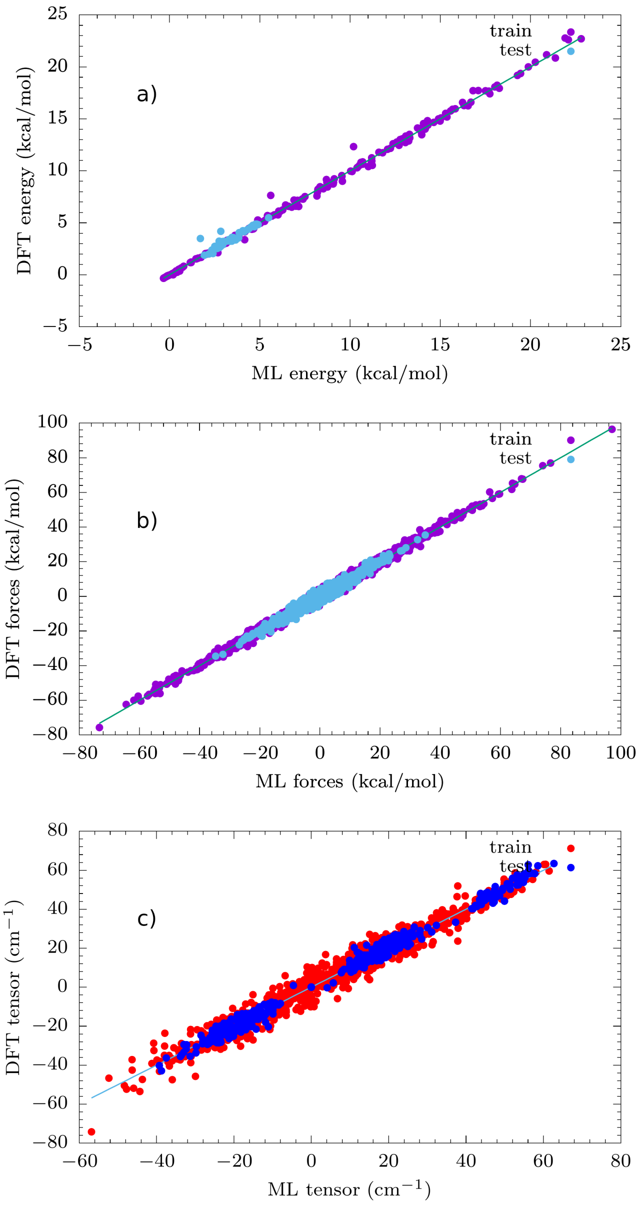

Figure S30: **Parity plots for predictions at T=75 K.** a) Energy, b) Forces, c) Tensorial components

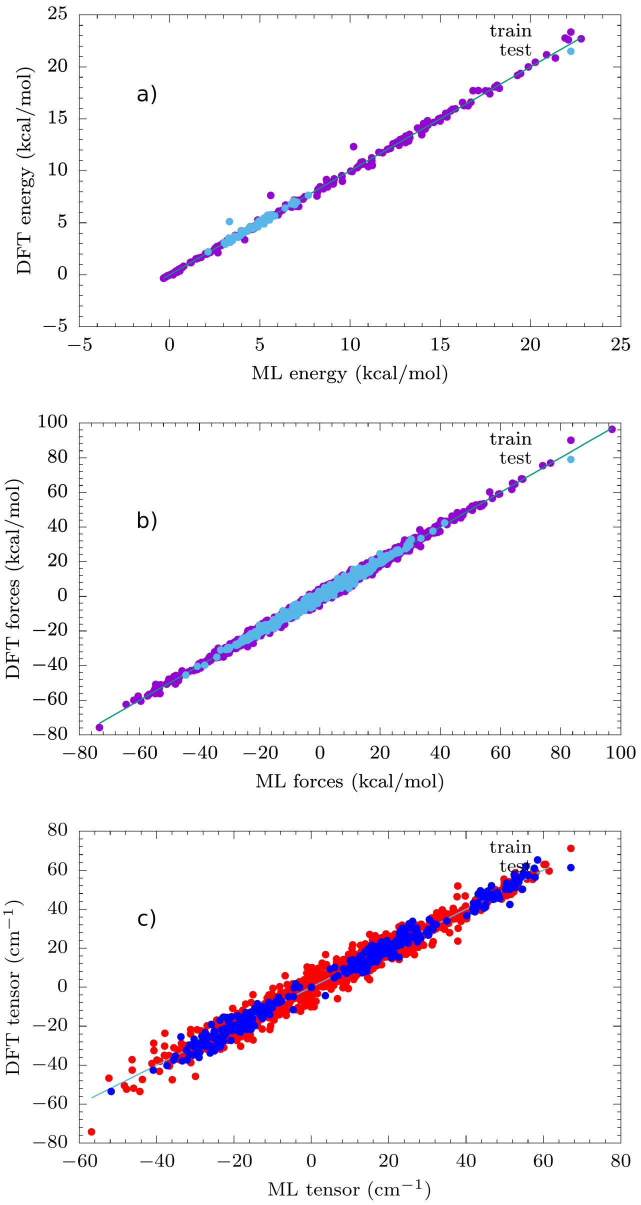

Figure S31: **Parity plots for predictions at T=100 K.** a) Energy, b) Forces, c) Tensorial components

4.2 Compound 2

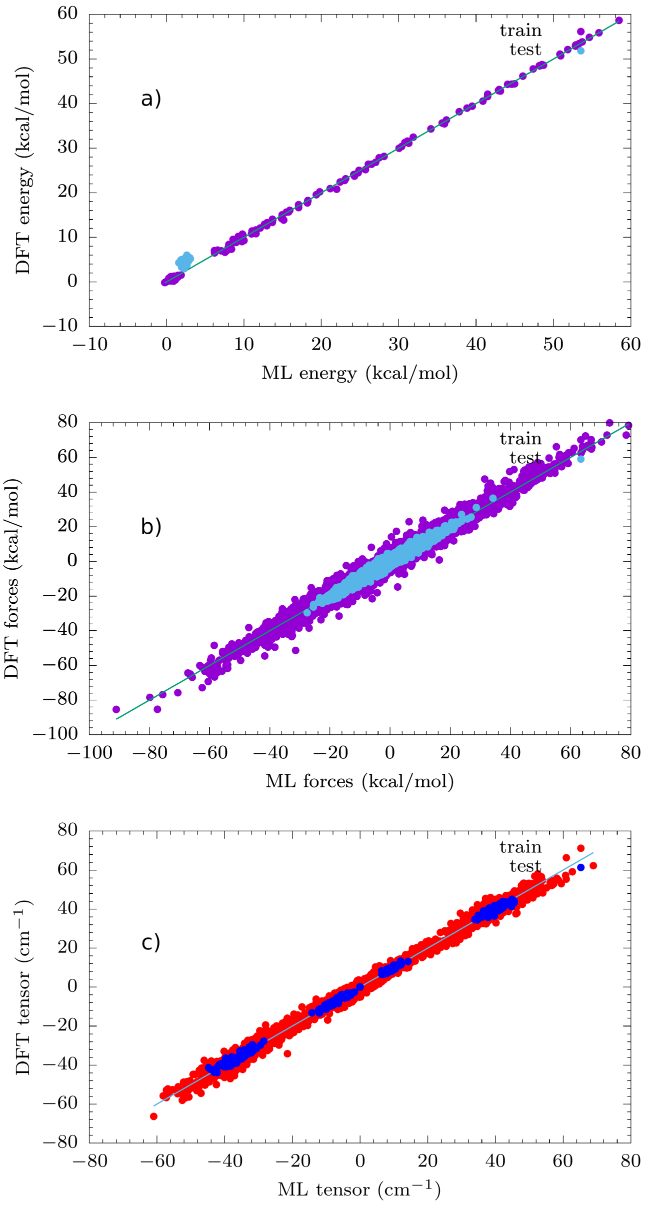

Figure S32: **Parity plots for predictions at T=25 K.** a) Energy, b) Forces, c) Tensorial components

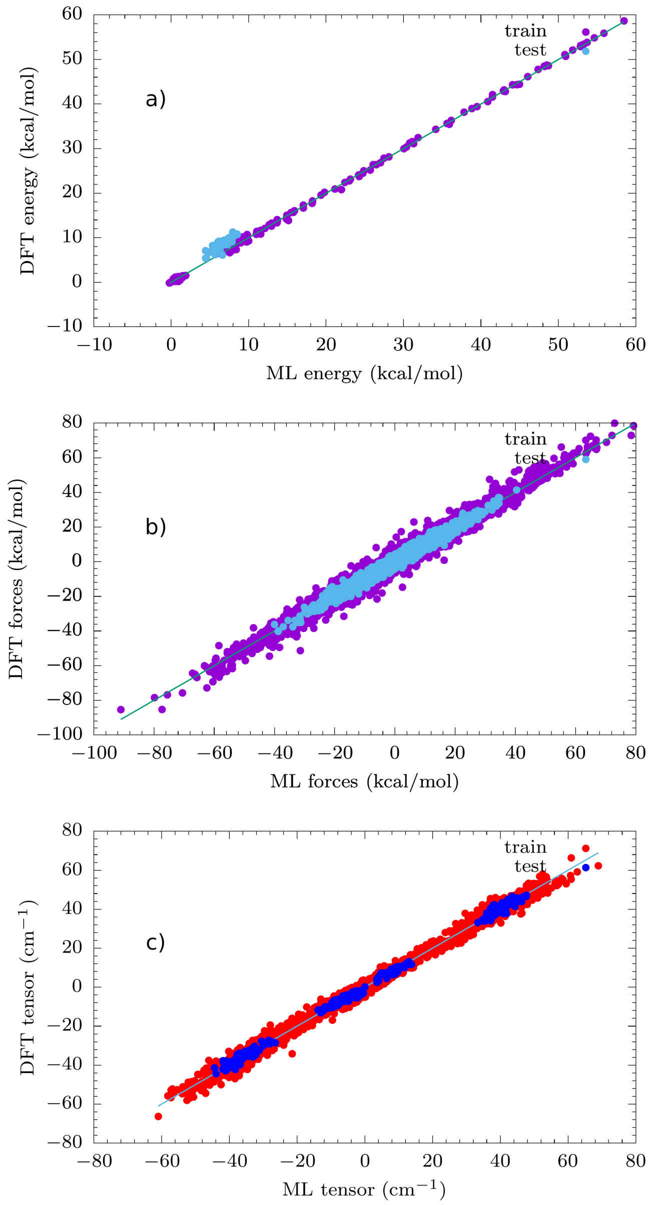

Figure S33: **Parity plots for predictions at T=50 K.** a) Energy, b) Forces, c) Tensorial components

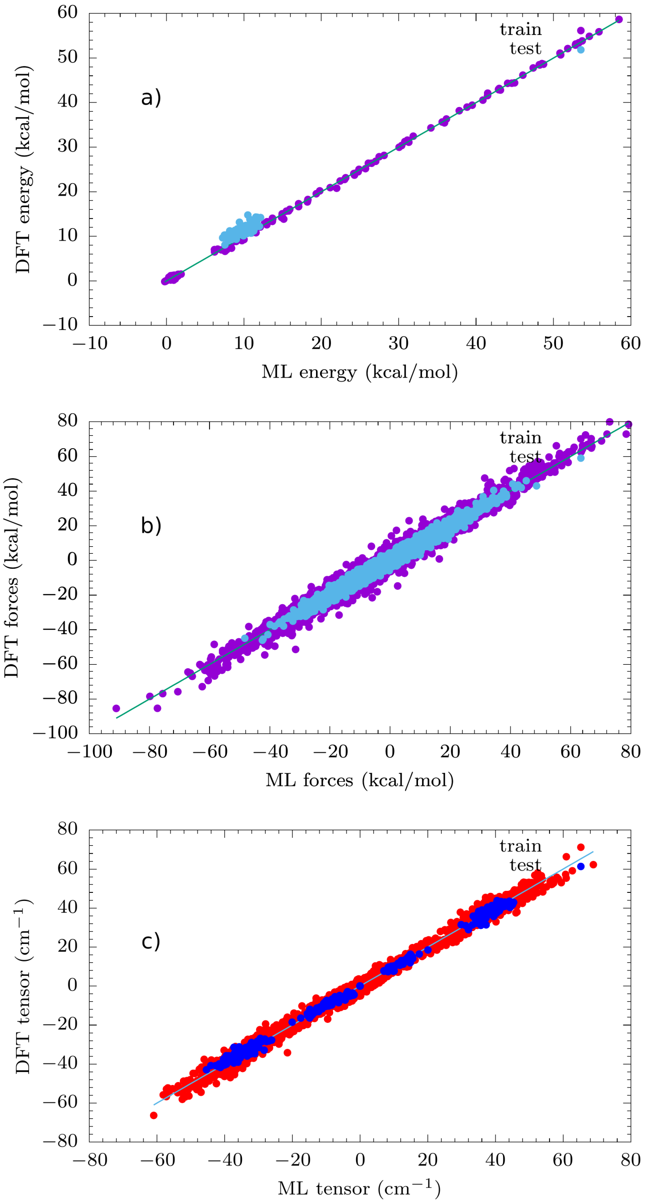

Figure S34: **Parity plots for predictions at T=75 K.** a) Energy, b) Forces, c) Tensorial components

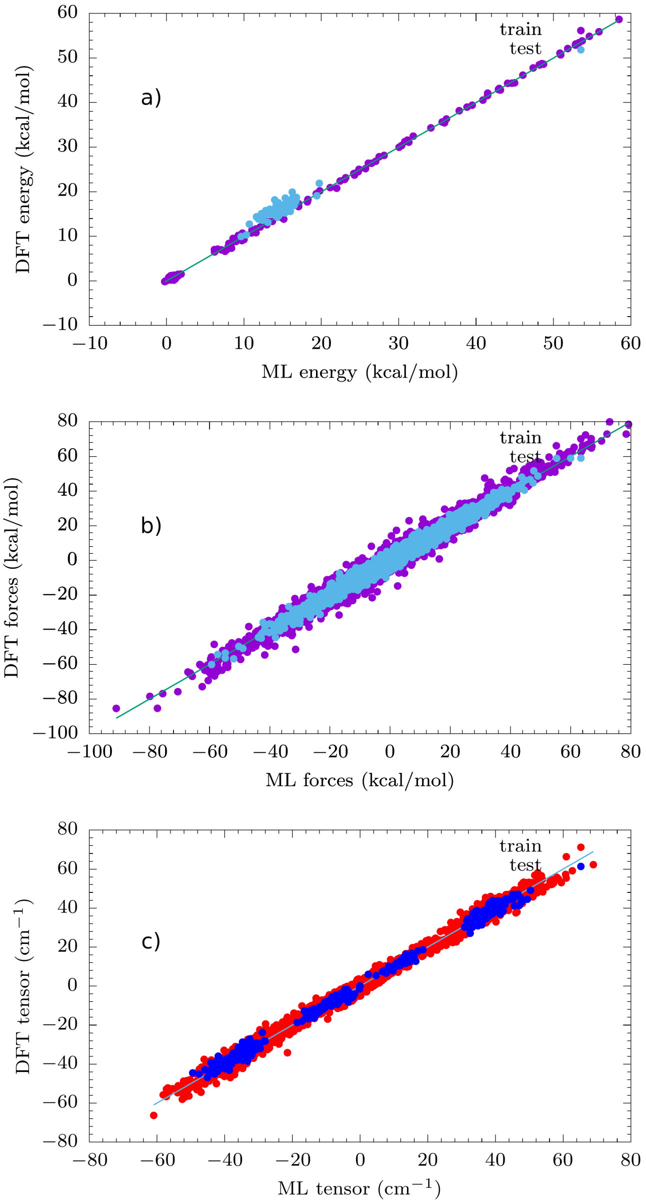

Figure S35: **Parity plots for predictions at T=100 K.** a) Energy, b) Forces, c) Tensorial components

4.3 Compound 3

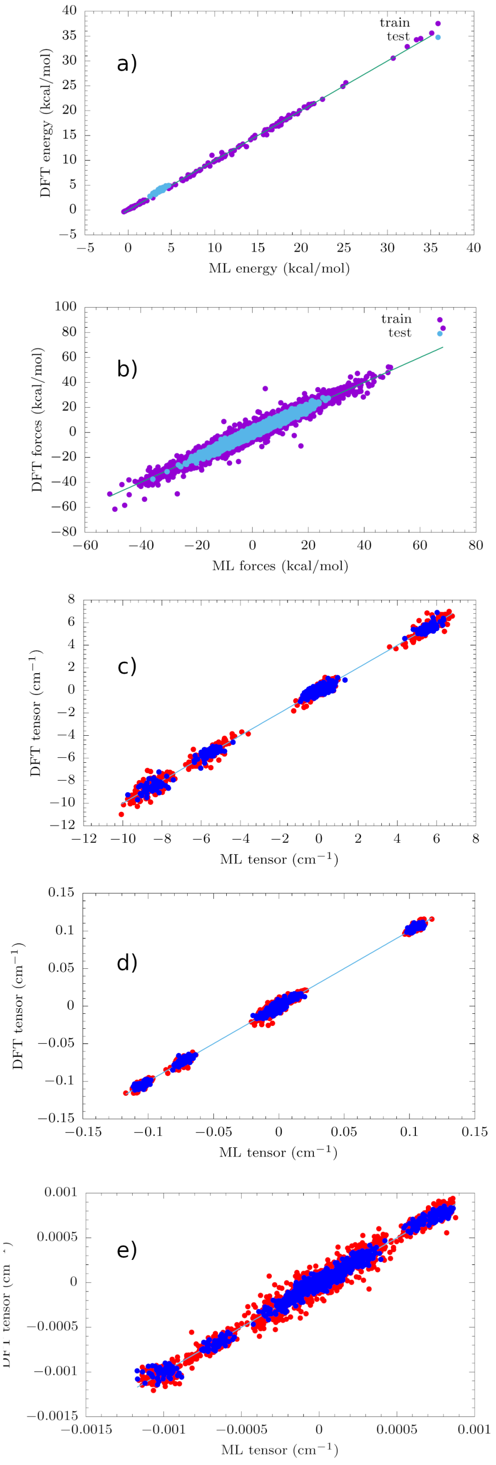

Figure S36: **Parity plots for predictions at T=25 K.** a) Energy, b) Forces, c) Tensorial components ($l = 2$), d) Tensorial components ($l = 4$),e) Tensorial components ($l = 6$)

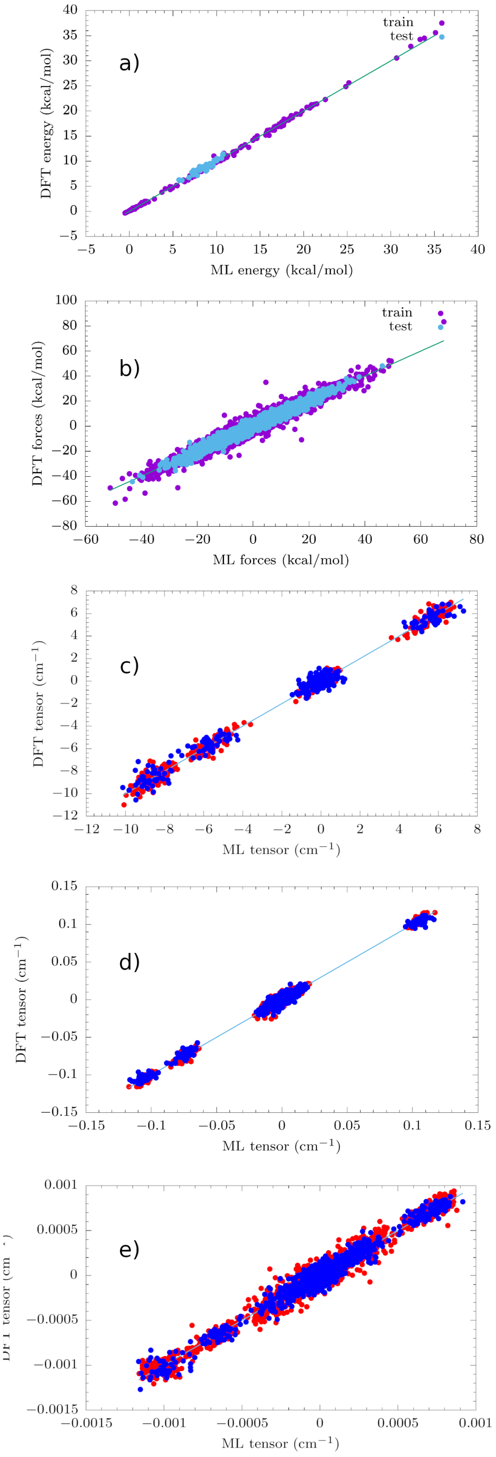

Figure S37: **Parity plots for predictions at T=50 K.** a) Energy, b) Forces, c) Tensorial components ($l = 2$), d) Tensorial components ($l = 4$),e) Tensorial components ($l = 6$)

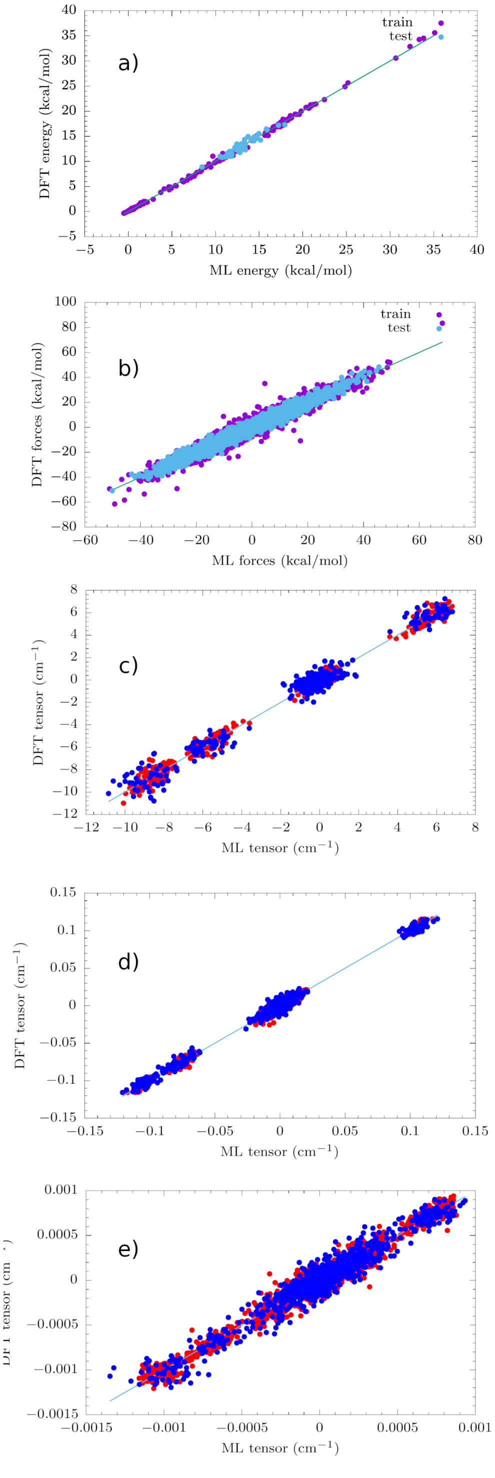

Figure S38: **Parity plots for predictions at T=75 K.** a) Energy, b) Forces, c) Tensorial components ($l = 2$), d) Tensorial components ($l = 4$),e) Tensorial components ($l = 6$)

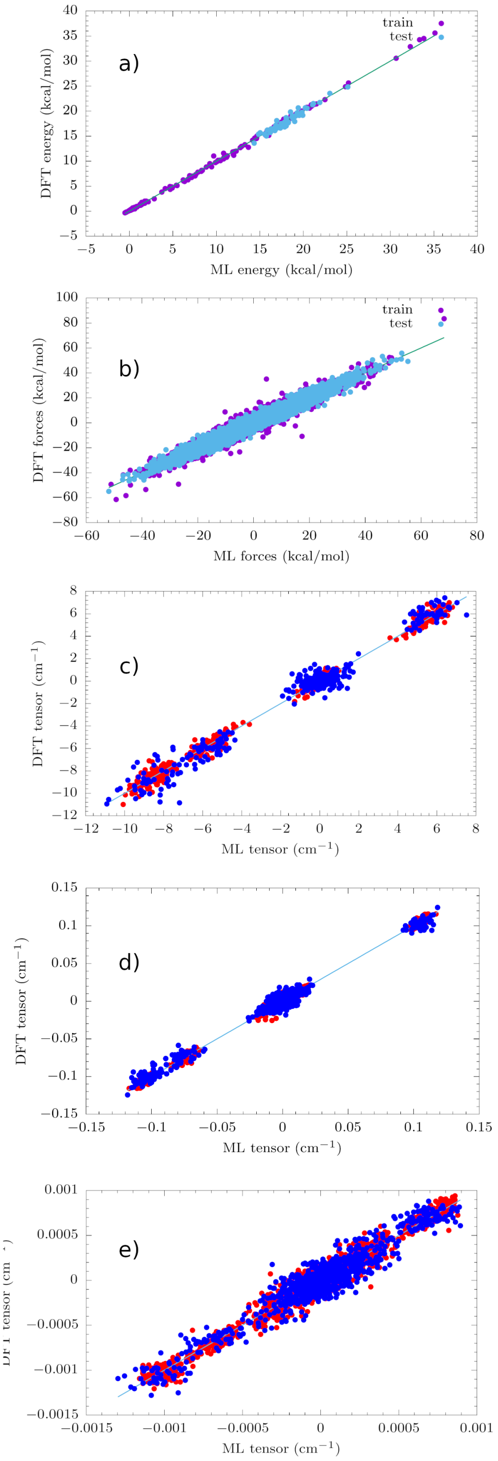

Figure S39: **Parity plots for predictions at T=100 K.** a) Energy, b) Forces, c) Tensorial components ($l = 2$), d) Tensorial components ($l = 4$),e) Tensorial components ($l = 6$)

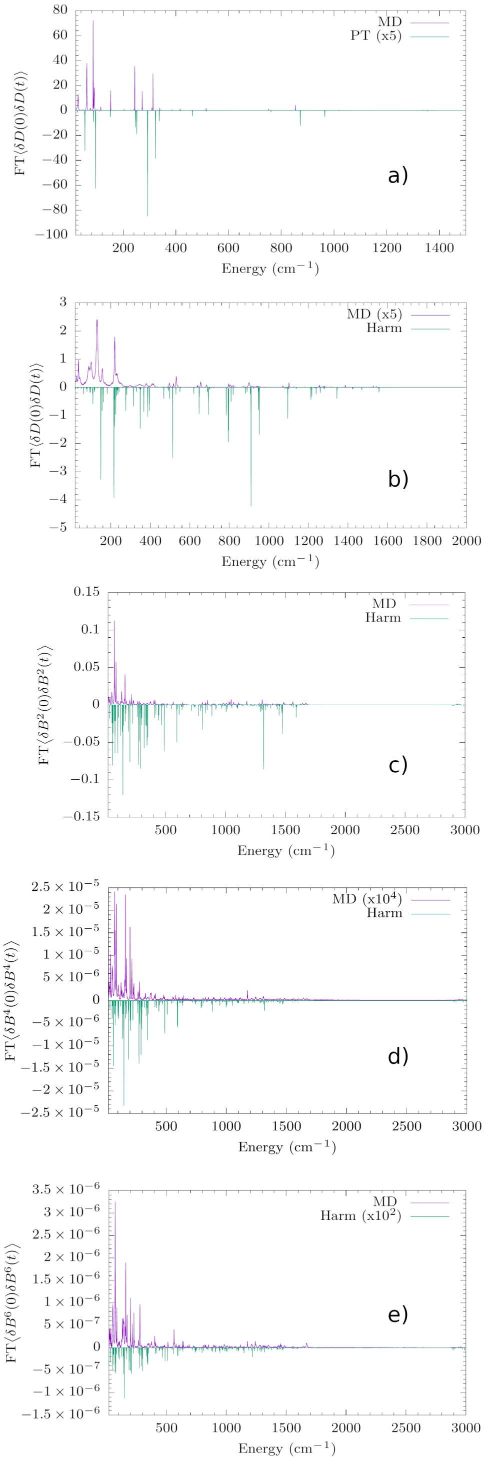

Figure S40: **Fourier transforms of the correlation functions at 25 K: comparison with the harmonic case**. a) Compound **1**, b) Compound **2**, c) Compound **3** ($l = 2$), d) Compound **3** ($l = 4$), e) Compound **3** ($l = 6$)

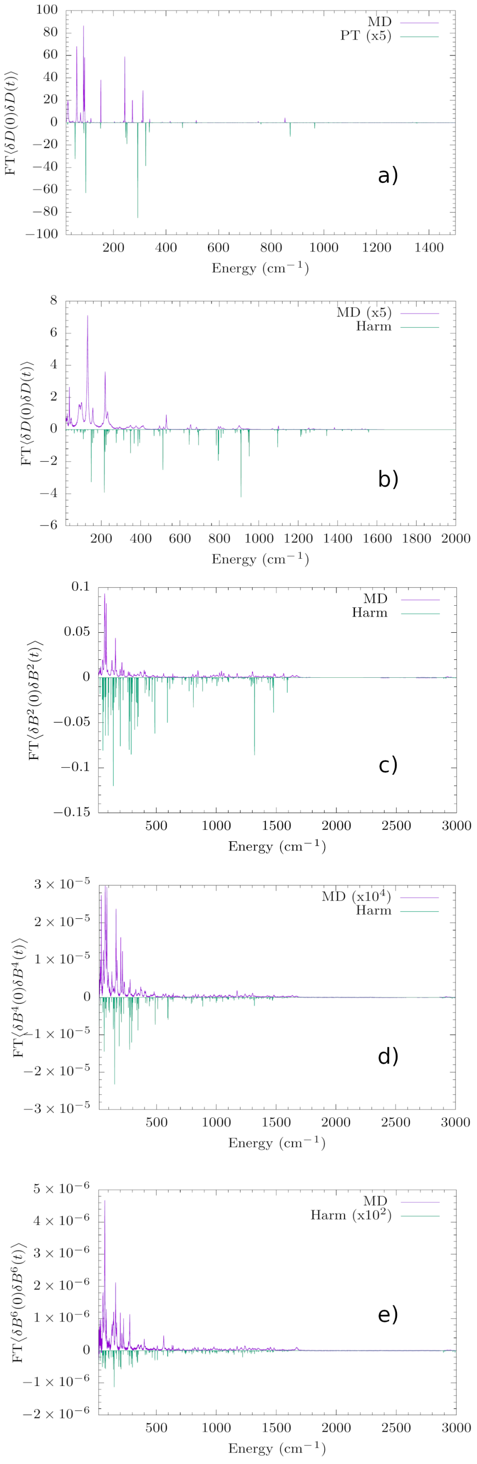

Figure S41: **Fourier transforms of the correlation functions at 50 K: comparison with the harmonic case**. a) Compound **1**, b) Compound **2**, c) Compound **3** ($l = 2$), d) Compound **3** ($l = 4$), e) Compound **3** ($l = 6$)

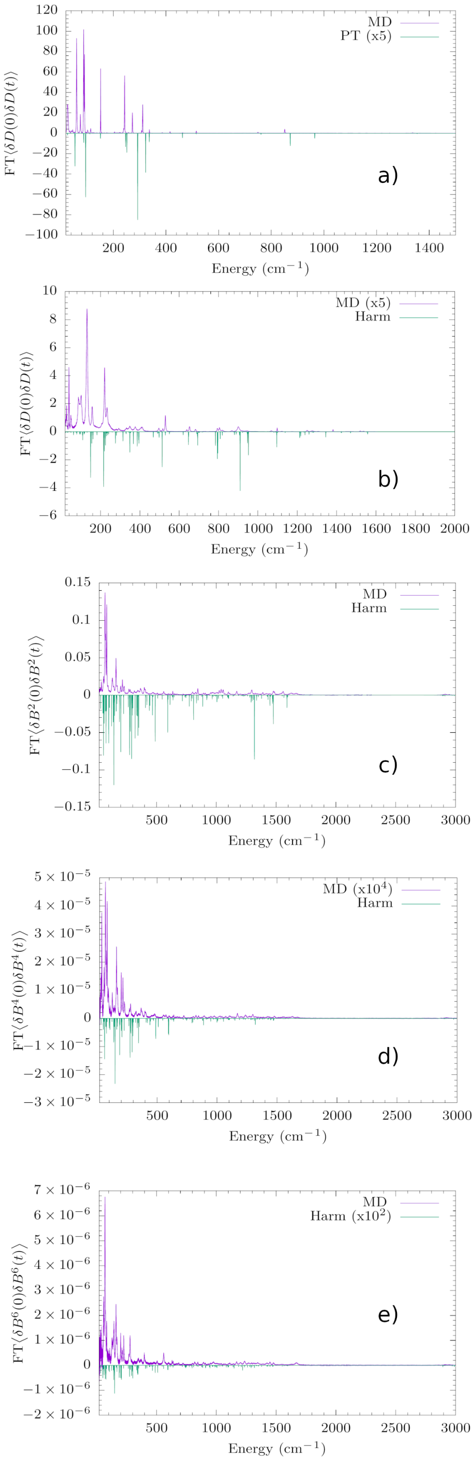

Figure S42: **Fourier transforms of the correlation functions at 75 K: comparison with the harmonic case**. a) Compound **1**, b) Compound **2**, c) Compound **3** ($l = 2$), d) Compound **3** ($l = 4$), e) Compound **3** ($l = 6$)

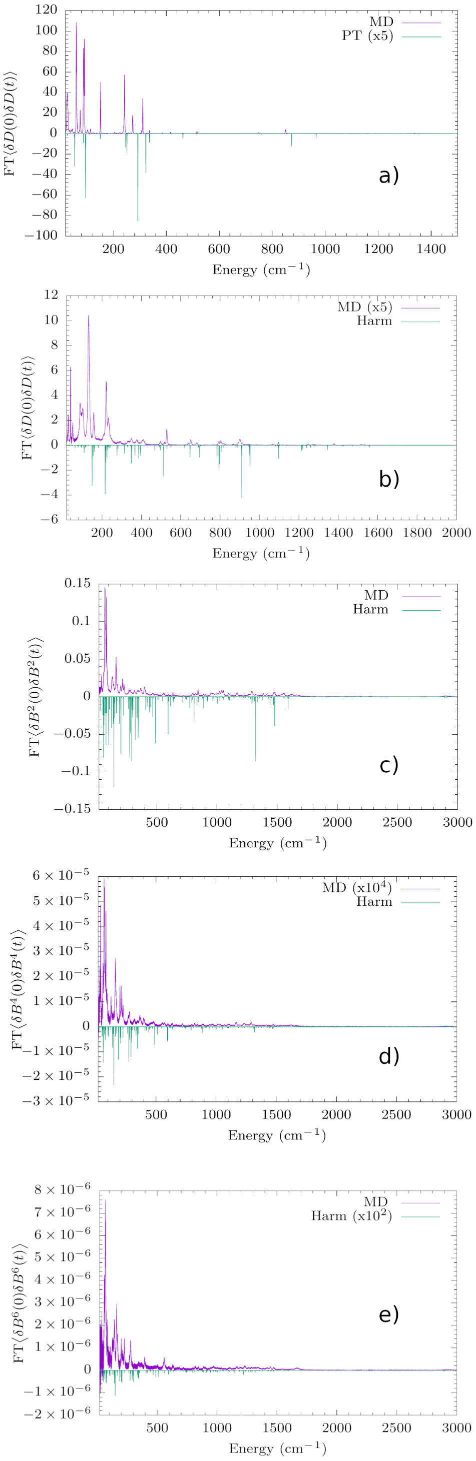

Figure S43: **Fourier transforms of the correlation functions at 100 K: comparison with the harmonic case**. a) Compound **1**, b) Compound **2**, c) Compound **3** ($l = 2$), d) Compound **3** ($l = 4$), e) Compound **3** ($l = 6$)

Table S4: **RMSE on training set for energies/forces and tensorial properties for MD trajectories at four different temperatures** The unit of measure is cm^{-1}. The results for **3** are reported for the different order of the tensor $l = 2, 4, 6$, in order. The training set size (TSS) is reported for the AL on energy/forces and in parentheses for the spin Hamiltonian tensors.

Comp	TSS	RMSE E	RMSE F	RMSE D/B
**1**	196(144)	0.30	0.87	3.62
**2**	157 (338)	0.38	1.68	2.23
**3**	167 (161)	0.30	1.76	0.26<br>$2.5\cdot10^{-3}$<br>$6.0\cdot10^{-5}$

Table S5: **RMSE on test set for energies/forces and tensorial properties for MD trajectories at four different temperatures.** The RMSE for energies and forces is reported in kcal/mol/ and kcal/mol/Å respectively; the RMSE for tensorial properties is reported in cm^{-1}. Temperatures of MD simulations are reported in K.

Compound	Temp	RMSE E	RMSE F	RMSE D or B
**1**	25	0.07	0.58	1.49
	50	0.23	0.73	1.91
	75	0.35	0.99	2.46
	100	0.30	0.82	2.90
**2**	25	2.38	1.17	0.97
	50	2.09	1.33	1.31
	75	2.02	1.48	1.47
	100	1.96	1.68	1.90
**3**	25	0.36	1.14	0.31 $2.7\cdot10^{-3}$ $5\cdot10^{-5}$
	50	0.44	1.60	0.50 $4.1\cdot10^{-3}$ $7.0\cdot10^{-5}$
	75	0.51	1.93	0.62 $4.6\cdot10^{-3}$ $9.7\cdot10^{-5}$
	100	0.57	2.24	0.74 $5.9\cdot10^{-3}$ $1.2\cdot10^{-4}$
